# SepT, a novel protein specific to multicellular cyanobacteria, influences peptidoglycan growth and septal nanopore formation in *Anabaena* sp. PCC 7120

**DOI:** 10.1128/mbio.00983-23

**Published:** 2023-08-31

**Authors:** Cristina Velázquez-Suárez, Benjamin L. Springstein, Mercedes Nieves-Morión, Andreas O. Helbig, Ann-Katrin Kieninger, Iris Maldener, Dennis J. Nürnberg, Karina Stucken, Ignacio Luque, Tal Dagan, Antonia Herrero

**Affiliations:** 1 Instituto de Bioquímica Vegetal y Fotosíntesis, CSIC and Universidad de Sevilla, Seville, Spain; 2 Institute of General Microbiology, Kiel University, Kiel, Germany; 3 AG Proteomics & Bioanalytics, Institute for Experimental Medicine, Christian-Albrechts-Universität zu Kiel, Kiel, Germany; 4 Department of Microbiology/Organismic Interactions, University of Tübingen, Tübingen, Germany; 5 Institute of Experimental Physics and Dahlem Centre of Plant Sciences, Free University of Berlin, Berlin, Germany; 6 Department of Food Engineering, Universidad de La Serena, La Serena, Chile; University of Nebraska-Lincoln, Lincoln, Nebraska, USA

**Keywords:** coiled-coil-rich proteins, divisome-dependent localization, filamentous cyanobacteria, septal peptidoglycan nanopores, septal proteins

## Abstract

*Anabaena* sp. PCC 7120 grows by forming filaments of communicating cells and is considered a paradigm of bacterial multicellularity. Molecular exchanges between contiguous cells in the filament take place through multiprotein channels that traverse the septal peptidoglycan through nanopores connecting their cytoplasms. Besides, the septal-junction complexes contribute to strengthen the filament. In search for proteins with coiled-coil domains that could provide for cytoskeletal functions in *Anabaena*, we identified SepT (All2460). SepT is characteristic of the phylogenetic clade of filamentous cyanobacteria with the ability to undergo cell differentiation. SepT-GFP fusions indicate that the protein is located at the cell periphery and, conspicuously, in the intercellular septa. During cell division, the protein is found at midcell resembling the position of the divisome. The bacterial adenylate cyclase two-hybrid analysis shows SepT interactions with itself and putative elongasome (MreB, RodA), divisome (FtsW, SepF, ZipN), and septal-junction (SepJ)-related proteins. Thus, SepT appears to rely on the divisome for localization at mature intercellular septa to form part of intercellular protein complexes. Two independently obtained mutants lacking SepT showed alterations in cell size and impaired septal and peripheral peptidoglycan incorporation during cell growth and division. Notably, both mutants showed conspicuous alterations in the array of nanopores present in the intercellular peptidoglycan disks, including aberrant nanopore morphology, number, and distribution. SepT appears, therefore, to be involved in the control of peptidoglycan growth and the formation of cell-cell communication structures that are at the basis of the multicellular character of this group of cyanobacteria.

**IMPORTANCE:**

Multicellular organization is a requirement for the development of complex organisms, and filamentous cyanobacteria such as *Anabaena* represent a paradigmatic case of bacterial multicellularity. The *Anabaena* filament can include hundreds of communicated cells that exchange nutrients and regulators and, depending on environmental conditions, can include different cell types specialized in distinct biological functions. Hence, the specific features of the *Anabaena* filament and how they are propagated during cell division represent outstanding biological issues. Here, we studied SepT, a novel coiled-coil-rich protein of *Anabaena* that is located in the intercellular septa and influences the formation of the septal specialized structures that allow communication between neighboring cells along the filament, a fundamental trait for the performance of *Anabaena* as a multicellular organism.

## INTRODUCTION

Bacterial multicellularity ranges from transient associations, such as colonies and biofilms, to permanent multicellular forms (1). The basic characteristics of multicellular prokaryotic organisms are mechanisms of cell-cell adhesion and intercellular communication (2). Additionally, bacterial multicellularity can be reinforced by processes of cell differentiation as in sporulating actinomycetes (3) and cyanobacterial trichomes (filaments of cells) (4). Cyanobacteria, which are generally classified as Gram-negative bacteria (5), are characterized by a large phenotypic diversity ranging from unicellular species to complex multicellular organisms, and some can undergo irreversible cell differentiation (6). Multicellular cyanobacteria that form trichomes and differentiate multiple cell types are considered the peak of prokaryotic complexity (7). Species of the Nostocaceae, including the model strain *Anabaena* sp. PCC 7120 (hereafter *Anabaena*), are characterized by the formation of linear trichomes, where semi-regularly interspaced heterocysts (cells specialized for nitrogen fixation) are produced upon combined nitrogen deprivation in a highly reproducible pattern (7, 8). Cells within a trichome are connected through incomplete segregation during cell division, which results in a common outer membrane and a shared periplasm that represents a communication conduit through the filament (9). Nonetheless, each cell is individually enclosed by a cytoplasmic membrane, whereas the septal peptidoglycan (PG) is continuous and engrossed in the intercellular regions (10 – 13).

In addition, *Anabaena* contains functional analogs to the eukaryotic gap junctions termed septal junctions, which are proteinaceous complexes located at the intercellular septa that connect the cells and facilitate intercellular communication along the filament (14 – 17). The septum-localized proteins, FraC, FraD, SepJ (17 – 19), SepI (20), and SepN (21), influence septum formation and cell-cell communication, and FraD and SepN could be localized to septal junctions by cryo electron tomography and are likely protein components of them (16, 21). The intercellular communication arrangement involves a nanopore array in the septal PG that has been considered to hold the septal-junction protein complexes. The formation of these structures depends on the activities of PG amidases of the AmiC type (22, 23) and the PG-binding protein SjcF1 (24). The importance of FraC, FraD, SepJ, and SepI for multicellularity in *Anabaena* is highlighted by a defect in trichome integrity and a resulting loss of multicellularity under diazotrophic growth conditions in mutant strains lacking any of these proteins (18 – 20). Notably, although diazotrophic growth requires FraC, FraD, SepJ, and SepI, heterocyst differentiation only depends on SepJ and SepI and not on FraC or FraD (18 – 20).

Cytoskeletal proteins are involved in essential tasks such as the determination of cell shape, cell division, and the organization of cell components. They all form protein polymers and are divided into three main classes: microtubules (represented by tubulin), microfilaments (represented by actin), and intermediate filaments. Although formerly thought to be specific to eukaryotes, homologs to the three classes of cytoskeletal proteins have also been universally found in bacteria, where they perform different cellular functions, some of which being non-cytoskeletal functions (25). In addition, coiled-coil-rich proteins (CCRPs) in bacteria also may form polymers and exert important structural functions such as the control of cell morphology, motility, cell division, and chromosome segregation [see reference (25)].

The bacterial actin homolog MreB is widespread in rod-shaped bacteria, where it is involved in the determination of the cell morphology (26). MreB, together with MreC, MreD, and PG synthases, constitutes the protein complex called the elongasome, which is responsible for cell elongation during cell growth (27, 28). MreB polymerizes, and it has been proposed that the coupled motion of MreB filaments about the long-cell axis coordinates PG-synthetic complexes and orients the circumferential insertion of new PG in the cylindrical part of the cell [see reference (29)]. In *Anabaena*, MreB is required for rod-shape determination but dispensable for cell viability (30, 31), although diazotrophic growth is compromised in its absence (31). Distinctly, besides in the cell periphery as in other rod-shaped bacteria, in *Anabaena* MreB, MreC, and MreD are localized to the divisome, influencing positioning of the Z-ring and septal PG-growth orientation, as well as in the intercellular mature septa, invoking a role in the formation of septal structures for intercellular communication (32, 33).

In most bacteria, the tubulin homolog FtsZ is essential for viability. FtsZ polymerizes into short filaments to form, together with other proteins, a ring at the future site of cell division, the so-called Z-ring. The Z-ring organizes the multiprotein complex termed the divisome, which includes the enzymatic machinery for PG growth to synthesize the new poles of the resulting daughter cells (34). In addition to FtsZ (35, 36), cyanobacteria possess the protein ZipN, which represents a central player in divisome assembly (37, 38). In *Anabaena*, ZipN is essential, and it has been identified as a principal tether of FtsZ to the cytoplasmic membrane and divisome organizer (39, 40). *Anabaena* also includes homologs for the divisome components FtsE, FtsK, FtsQ, FtsX, FtsI, FtsW, and SepF [see references (40 – 42)].

In *Anabaena*, the septal-junction-related proteins, SepJ, which itself contains an N-terminal coiled-coil domain and forms multimers (43, 44), FraC (18), and SepI (20) are recruited to the intercellular septa through interactions with the divisome during cell division, thus providing a link between cell division and cell-cell communication. In addition, other cyanobacterial CCRPs were identified more recently to perform cytoskeletal and cytoskeletal-like functions in a diverse set of cyanobacterial species (45), including the heteropolymer-forming ZicK and ZacK (46).

In this work, we aimed at the characterization of another coiled-coil-rich protein and deciphering its function in *Anabaena* as a model of bacterial multicellularity. We found that All2460, which we have termed SepT, is a new element connecting cell division and PG growth to the formation of the septal structures involved in cell-cell adhesion and intercellular communication through the filament.

## RESULTS

### All2460 predicted domains and phylogenetic distribution

**Fig 1:**
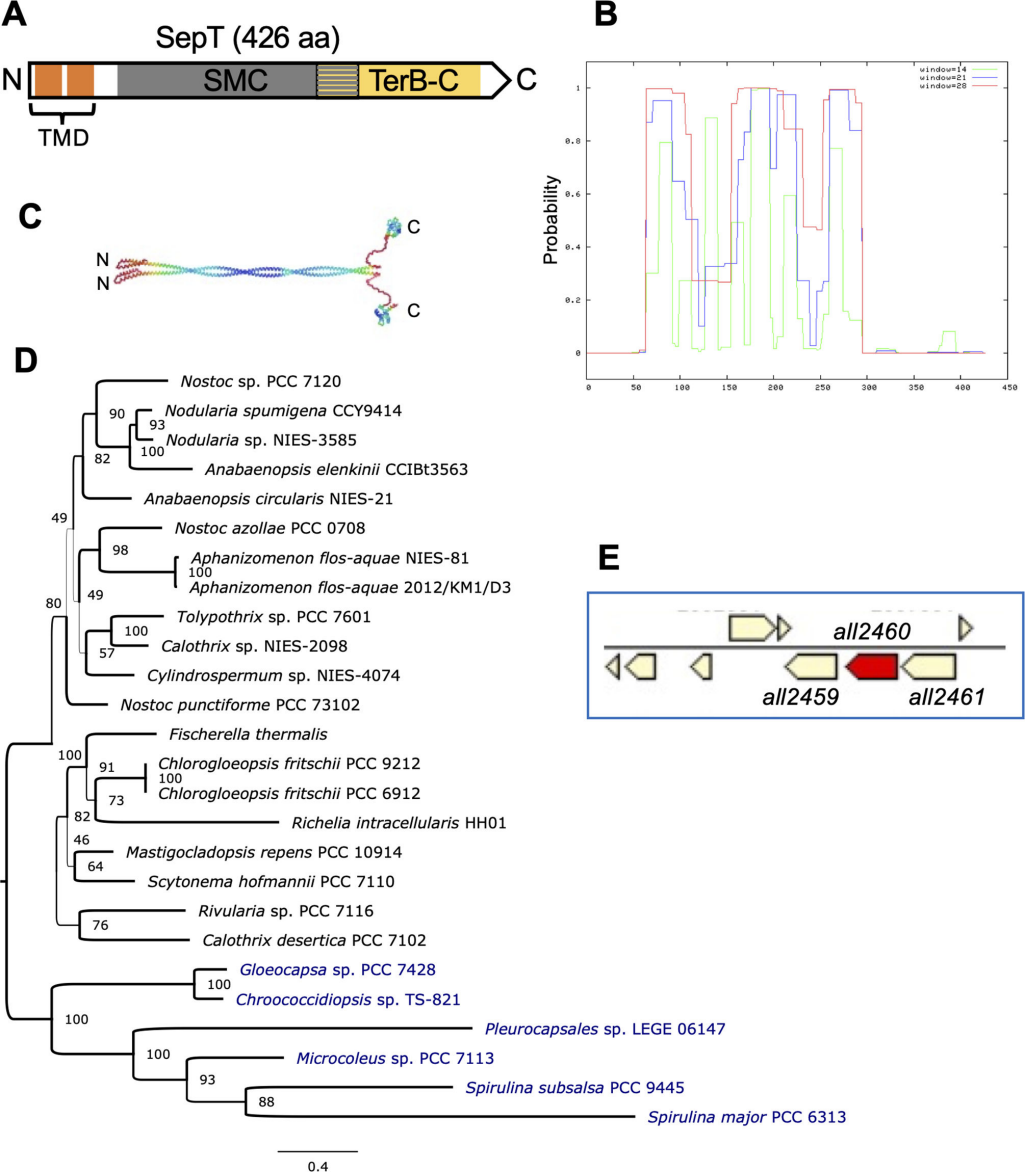
SepT domain distribution and phylogeny. (**A**) Prediction of conserved domains in SepT using the Conserved Domain Search (NCBI). Orange bars indicate the presence of the two transmembrane domains; a structural maintenance of chromosomes domain is depicted in gray, and a TerB-C domain (putative metal chelating) domain is shown in yellow. The SMC and TerB-C domains overlap in part, which is highlighted by a mixed gray-yellow part. (**B**) Prediction of coiled-coil-rich regions using the COILS algorithm and three different screening windows for repetitive heptamer sequences (windows 14, 21, and 28). (**C**) Prediction of the structure of a SepT dimer (colored by pLDDT) according to AlphaFold algorithm. (**D**) Phylogenetic tree of selected SepT homologs. Heterocyst-forming species are written in black, and non-heterocystous species are written in blue. *Nostoc* sp. PCC 7120 is the same strain as *Anabaena* sp. PCC 7120. Bootstrap values are shown next to ancestral node, and the branch width is scaled to the bootstrap values. (**E**) The genomic region of *all2460* taken from the Integrated Microbial Genomes and Microbiomes of the Joint Genome Institute database.

Initially selected in a screen, using COILS (47), searching for coiled-coil-rich proteins with a putative function in the *Anabaena* multicellularity and morphology (45), All2460 is characterized by three distinct coiled-coil-rich regions as well as two N-terminal transmembrane domains (TMDs, residues 12–50, predicted with TMHMM) (Fig. 1A and B). Using the Conserved Domain Search (CD Search, NCBI), All2460 is predicted to contain a structural maintenance of chromosomes (SMC) and a TerB-C domain, suggesting a metal-dependent function in chromosome biology (Fig. 1A). Predictions of the putative localization of the C-terminal non-TMD parts consistently placed the C-terminus in the cytoplasm (predicted using PSIPRED, PSORTb, and Gneg-mPLoc). Using AlphaFold (48), All2460 is predicted to form a homodimer (Fig. 1C). A further search for All2460 homologs using amino acid sequence similarity shows that all tested filamentous heterocyst-forming cyanobacteria encode a protein homologous to All2460 (Data Set S1; Fig. S1; Fig. 1D). Outside the heterocyst formers, there are some homologs that using our thresholds are clustered in the same protein family as All2460. These homologs are found in filamentous non-heterocystous cyanobacteria such as *Spirulina*, and closely related genera to the order Nostocales such as *Gloeocapsa*, *Chroococcidiopsis,* or *Pleurocapsa* (49). Moreover, the protein sequence alignment of SepT homologs (Fig. S1) indicates a high-sequence divergence between homologs from the heterocyst-forming and non-heterocystous cyanobacteria, suggesting also functional divergence.

To further study the genomic neighborhood of All2460, we searched for the presence of conserved gene order at the All2460 locus using CSBFinder-S (50). Our results reveal a conserved synteny block that includes two genes next to *all2460*, namely *all2459* and *all2461* (Fig. 1E). Unlike All2460, both All2459 and All2461 are not predicted to contain TMDs but both are predicted to contain P-loop NTPase domains, which are commonly found in MinD, ParA, and other DNA partitioning systems (51). All three genes are about twofold upregulated 21 hours after nitrogen stepdown (52), suggesting some involvement in diazotrophic growth.

### All2460 localization in *Anabaena*

**Fig 2:**
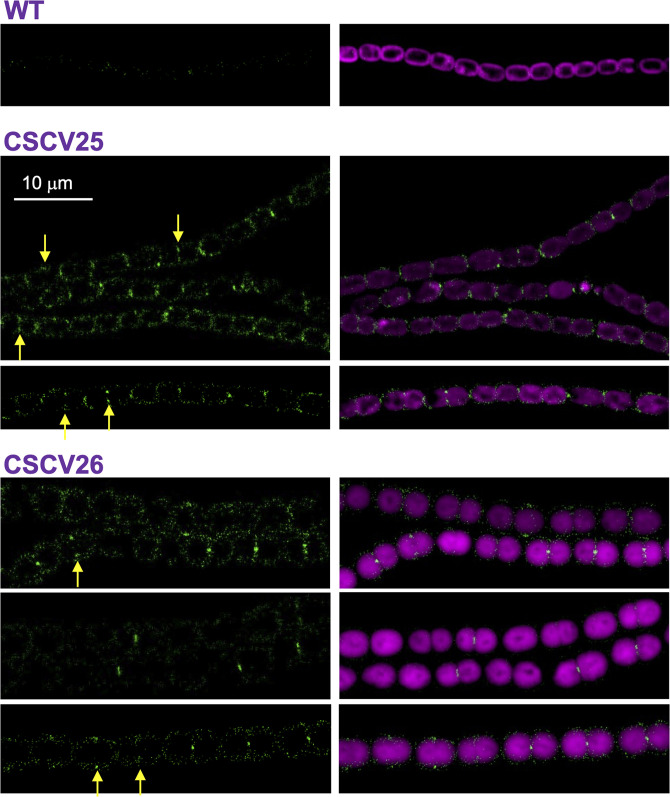
Localization of SepT-GFP in *Anabaena* PCC 7120 and BS1 mutant. Strains *Anabaena* WT, CSCV25 (expressing SepT-GFP in the wild-type background), and CSCV26 (expressing SepT-GFP in BS1 background) were grown in and transferred to BG11 medium at a cell density corresponding to 0.5 µg Chl mL^−1^ and incubated under culture conditions. After 24 hours, filaments were observed by confocal microscopy with a Fluoview equipment. GFP fluorescence (green) and merged GFP and cyanobacterial autofluorescence (magenta) images are shown. Yellow arrows point to GFP fluorescence matching the divisome. Magnification is the same for all micrographs.

We studied the localization of All2460 in *Anabaena* by means of fusions to GFP. For that, a replicative plasmid encoding All2460 C-terminally fused to GFP, directed by the native promoter sequence, was transferred to *Anabaena* wild type (WT), generating strain CSCV25, and to strain BS1 (Δ*all2460*) (see below), generating strain CSCV26. In both strains, CSCV25 and CSCV26, GFP fluorescence was detected in the cell periphery and, conspicuously, in the intercellular septa between contiguous cells along the filament (Fig. 2). Remarkably, midcell GFP bands were detected in some dividing cells, matching the localization of the progressing new septum. When the N-terminal transmembrane domain of SepT was deleted, the resulting protein fused to GFP produced fluorescence in patches without any specific localization in the cell (Fig. S2), suggesting that membrane anchorage is essential for the correct localization. These observations suggest that All2460 is a new septal protein of *Anabaena,* and accordingly, we term it SepT.

### Analysis of SepT interactions

**Fig 3:**
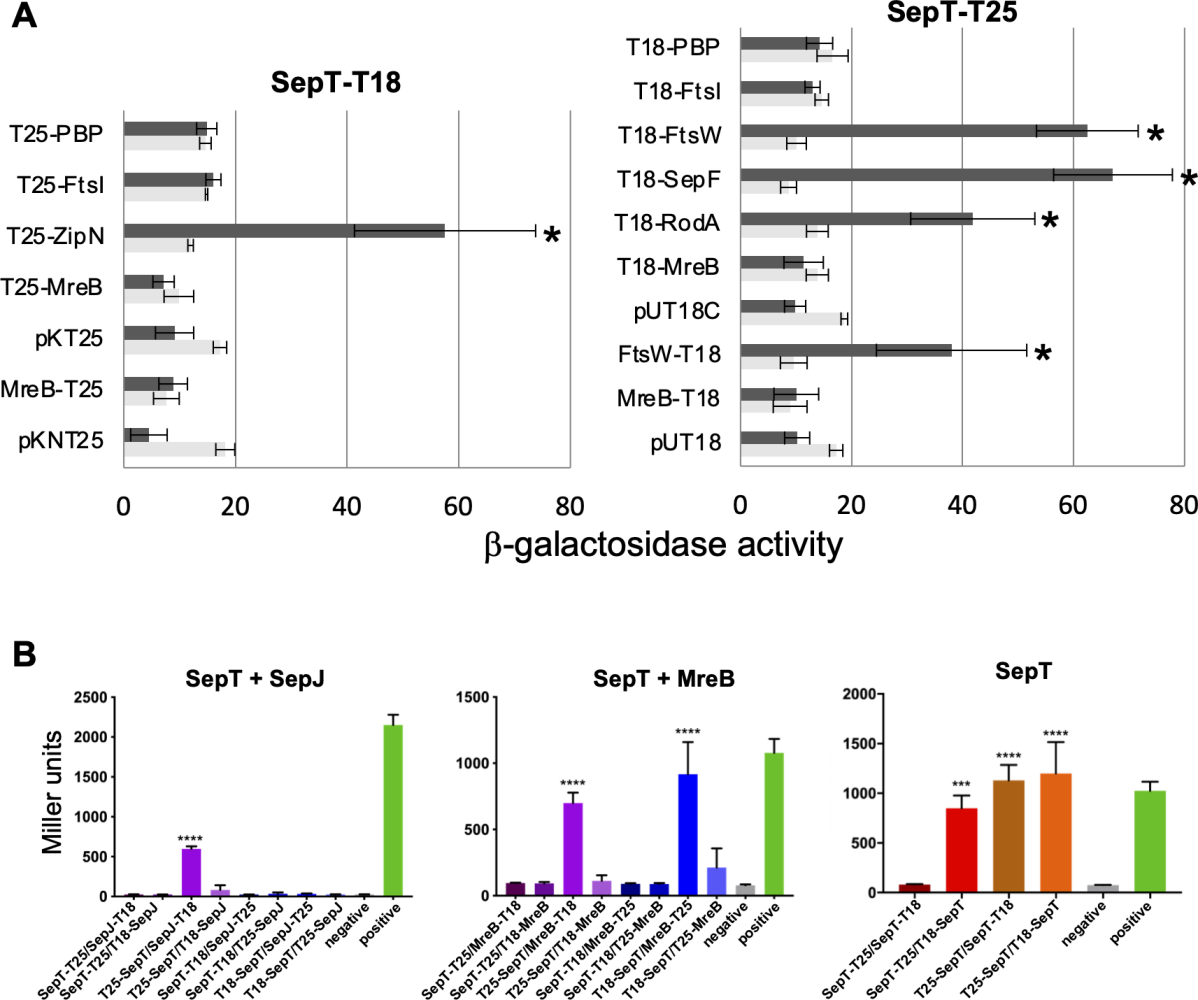
Analysis of SepT interactions by BACTH. The topology of each fusion is indicated by the order of components (T18-protein and T25-protein denote the corresponding adenylate cyclase domain fused to the N-terminus of the tested protein, whereas protein-T18 and protein-T25 denote fusions to the C-terminus). (**A**) Interaction of protein pairs produced in *Escherichia coli* was assayed by measurements of β-galactosidase activity (nmol ONP min^−1^ mg protein^−1^) in liquid cultures incubated at 30°C. Data are the mean and standard deviation of two to nine determinations of the activity with the indicated protein fused to T25 (or the empty vectors pKNT25 or pKT25) and SepT-T18 (dark bars), or the indicated protein (or the empty vectors pKNT25 or pKT25) and pUT18C (clear bars); or with the indicated protein fused to T18 (or the empty vectors pUT18C or pUT18) and SepT-T25 (dark bars), or the indicated protein (or the empty vectors pUT18C or pUT18) and pKT25 (light bars). Significance of differences was assessed by Student’s *t-*tests. Asterisks indicate strains expressing a pair of tested proteins that exhibited β-galactosidase activity significantly different (*P* < 0.023) from the two controls: the strains expressing each fused protein and containing the complementary empty vector. (**B**) *E. coli* cells were subjected to β-galactosidase assay in triplicates from three independent colonies after grown in liquid cultures at 20°C for 2 days. Quantitative values are given in Miller units, and the mean results from three independent colonies are presented. Negative: N-terminal T25 fusion construct of the respective protein co-transformed with empty pUT18C. Positive: Zip/Zip control. Error bars indicate standard deviations (*n* = 3). Values indicated with asterisks are significantly different from the negative control. ***P < 0.001, ****P < 0.0001 (Dunnett’s multiple comparison test and one-way ANOVA).

The apparent localization of SepT to midcell, the intercellular septa, and the cell periphery drove us to investigate its possible interactions with known or putative elements of the *Anabaena* divisome, the septal junctions or the elongasome. For this purpose, we used the bacterial adenylate cyclase two-hybrid (BACTH) system. Regarding divisome components, we tested interactions of SepT with FtsZ, ZipN, Alr0487 (putative SepF), All0154 (putative PG glycosyl-transferase, FtsW), and Alr0718 (PG transpeptidase, FtsI). Strong interactions were detected for the pairs SepT-T18/T25-ZipN, SepT-T25/T18-SepF, SepT-T25/T18-FtsW, and SepT-T25/FtsW-T18 (Fig. 3A). Regarding septal proteins, we tested FraC, FraD, SepJ, and SepI. Significant, although comparatively low, interaction was detected only with SepJ (Fig. 3B). Regarding elongasome components, we tested interactions of SepT with MreB, MreC, MreD, Alr0653 (putative PG glycosyl-transferase, RodA), and Alr5045 (elongasome transpeptidase). Interactions were detected between SepT-T25 and T18-RodA (Fig. 3A), between T25-SepT and MreB-T18, and between T18-SepT and MreB-T25 (Fig. 3B). Finally, SepT was detected to be able of self-interactions (Fig. 3B).

To identify further interaction partners of SepT, we performed co-immunoprecipitations of *Anabaena* WT expressing SepT-GFP and analyzed co-precipitated proteins by LC-MS/MS. With this approach, we were able to verify the interaction of SepT with MreB and SepJ. Additionally, proteins that co-precipitated with SepT included All4981 and All2459, from its own synteny block, and three different penicillin-binding proteins (PBPs): All2981, All2952, and Alr0718 (the full list of interactions identified is supplied in Data Set S2).

### Inactivation of *sepT*

**Fig 4:**
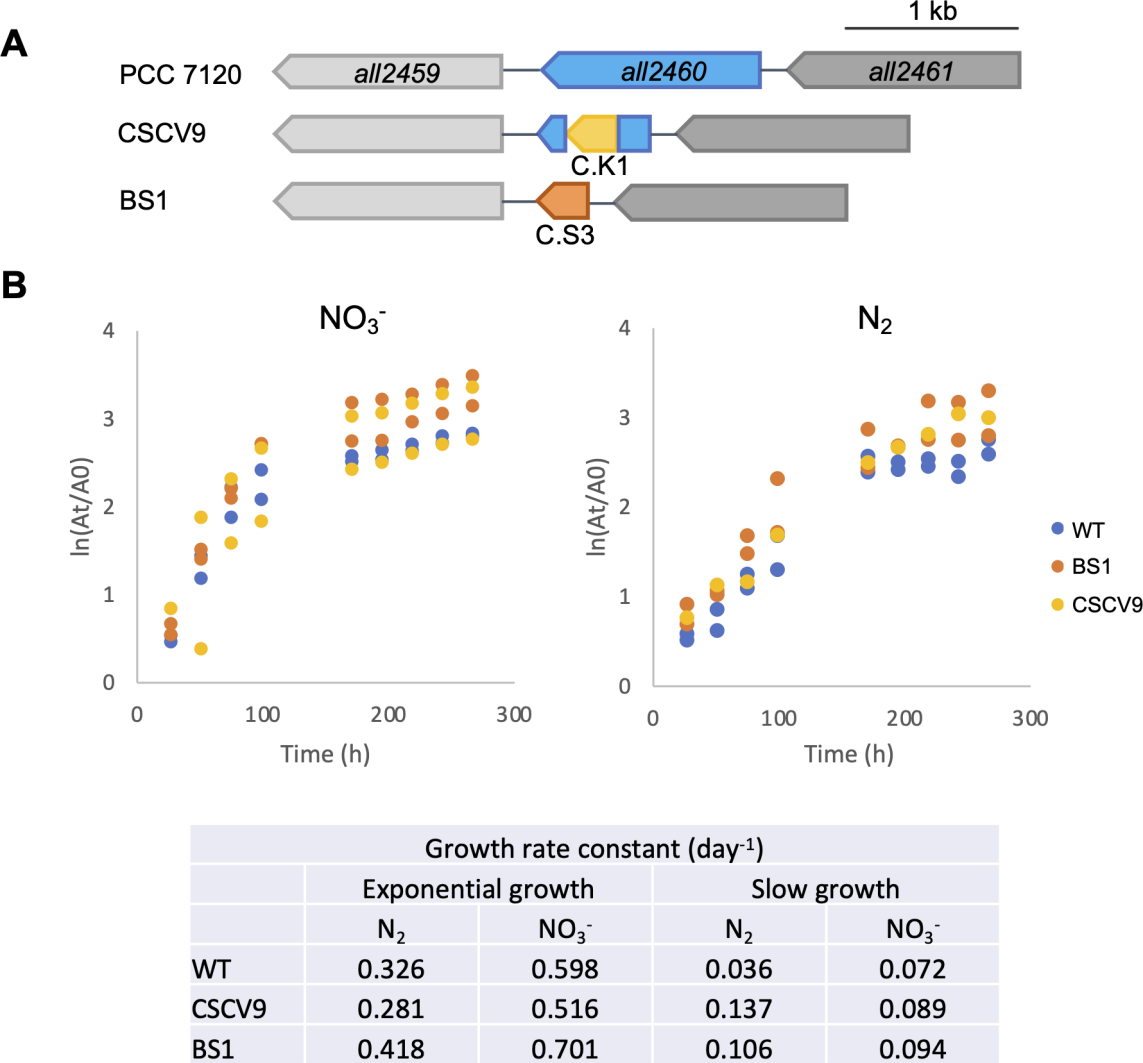
Genomic structure and growth of strains with inactivated *sepT*. (**A**) Schematic of the genetic structure of strains CSCV9 and BS1 in the *sepT* genomic region. (**B**) Filaments of strains *Anabaena* WT, CSCV9, and BS1 were grown in BG11 medium, transferred to BG11 (containing NaNO_3_^−^) or BG11_0_ (no combined nitrogen) at a cell density corresponding to 0.5 μg Chl mL^−1^, and incubated under culture conditions. At the indicated times, the OD_750_ (At) was measured in aliquots of each culture. The values of two independent cultures of each condition (one culture of CSCV9 under N_2_) were represented and adjusted to sequential linear functions. A0 represents the OD_750_ at the start of the culture. Growth rate constant, μ (day^−1^), corresponds to ln2/*t*_*d*_, where *t*_*d*_ is the doubling time, calculated from the increase in OD_750_ from 0 to 98.5 hours (exponential growth) and from 170.5 to 266.5 hours (slow growth) of incubation as above. Mann-Whitney tests indicated no significance of differences (*P* > 0.1) between each mutant and the WT for any time and condition.

**Fig 5:**
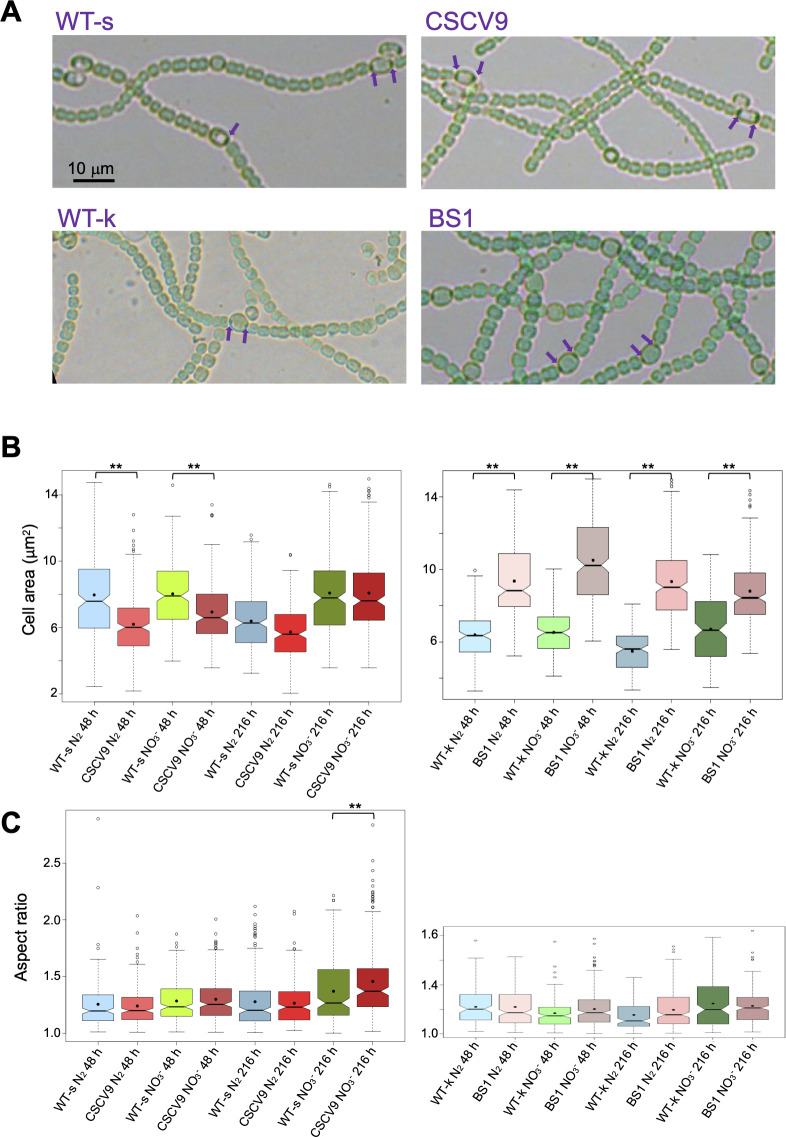
Cell size and morphology of *sepT* mutants. Filaments of *Anabaena* WT (WT-s, the parental for CSCV9; WT-k, the parental for BS1), CSCV9 (*sepT*::C.K1), and BS1 (*sepT*::C.S3) grown in BG11 medium were transferred to BG11 or BG11_0_, at a cell density corresponding to 0.5 µg Chl mL^−1^, and incubated under culture conditions. (**A**) After 48 hours, filaments from BG11_0_ cultures were photographed. Purple arrows point to polar granules in heterocysts. Magnification is the same for all micrographs. (**B and C**) After 48 and 216 hours, aliquots of each culture were photographed and used for cell area (**B**) and aspect ratio (**C**) determinations. Two hundred to three hundred cells (vegetative cells in the diazotrophic cultures) from two different cultures of each strain and condition (150 cells for WT-k) were measured. The aspect ratio is the result of dividing the length of the axis parallel to the filament by the length of the axis perpendicular to the filament. Notched boxplot representations of the data are shown. The mean values are represented by black dots. Significant differences (*P* < 0.01), assessed by Student’s *t*-tests, are indicated by **.

To study the function of SepT, we sought the generation of *Anabaena* derivatives lacking a functional *all2460* ORF. Strain CSCV9, generated in the Seville lab, bears gene-cassette C.K1, encoding resistance to kanamycin and neomycin, inserted into *all2460* (Fig. 4A). Growth of this mutant was studied in media supplemented with nitrate or lacking combined nitrogen (Fig. 4B). It was able to grow under the two conditions, exhibiting growth rates similar to those of *Anabaena* WT both in the exponential growth phase and in the phase of slow growth (Fig. 4B). Consistent with these results, strain BS1, which was previously generated in the Kiel lab, bearing gene cassette C.S3 substituting for *all2460* (Fig. 4A), also exhibited growth rates comparable to those of *Anabaena* WT (Fig. 4B). In the absence of combined nitrogen, both CSCV9 and BS1 exhibited mature heterocysts with conspicuous polar granules, as is the case for *Anabaena* WT (Fig. 5A).

The cell area and, as an estimation of the cell morphology, the aspect ratio (the ratio between the cell axis parallel to the filament and the axis perpendicular to the filament) were determined in strains CSCV9 and BS1. Because strains maintained in different labs have frequently been noticed to exhibit some different phenotypic characteristics, each of the two mutants was compared to the respective *Anabaena* WT strain from which they were derived (WT-s, WT from Seville; WT-k, WT from Kiel). While actively growing cells (48 hours) of CSCV9 were smaller than those of its parental strain in the presence and absence of combined nitrogen (Fig. 5A and B) (Student’s *t*-tests indicate significant differences, *P* < 0.01, for comparison of WT-s and CSCV9 with NO_3_^–^ or N_2_ after 48 hours of incubation; and non-significant differences, *P* > 0.05, after 216 hours), cells of BS1 appeared larger than those of its parental strain (Fig. 5A and B). (Student’s *t*-tests indicate significant differences, *P* < 0.01, for comparison of WT-k and BS1 with NO_3_^–^ or N_2_ after 48 or 216 hours of incubation.) The alterations in cell size in CSCV9 and BS1 suggest that both strains suffer a certain discoordination between cell growth and division. In contrast to C.K1 introduced in CSCV9, the C.S3 gene cassette introduced in BS1 bears strong transcriptional terminators, which could affect the expression of the downstream ORF *all2459*. This could contribute to explain the differences in the effects of the mutations in CSCV9 and BS1.

Finally, cell aspect ratio determinations indicated that the morphology of strains CSCV9 and BS1 is similar to that of their respective parentals (Fig. 5C). (Student’s *t*-tests indicate significant differences, *P* = 0.002, only for comparison of CSCV9 and its WT after 216 hours of incubation with NO_3_^–^.)

### Localization of PG growth in *sepT* mutants

**Fig 6:**
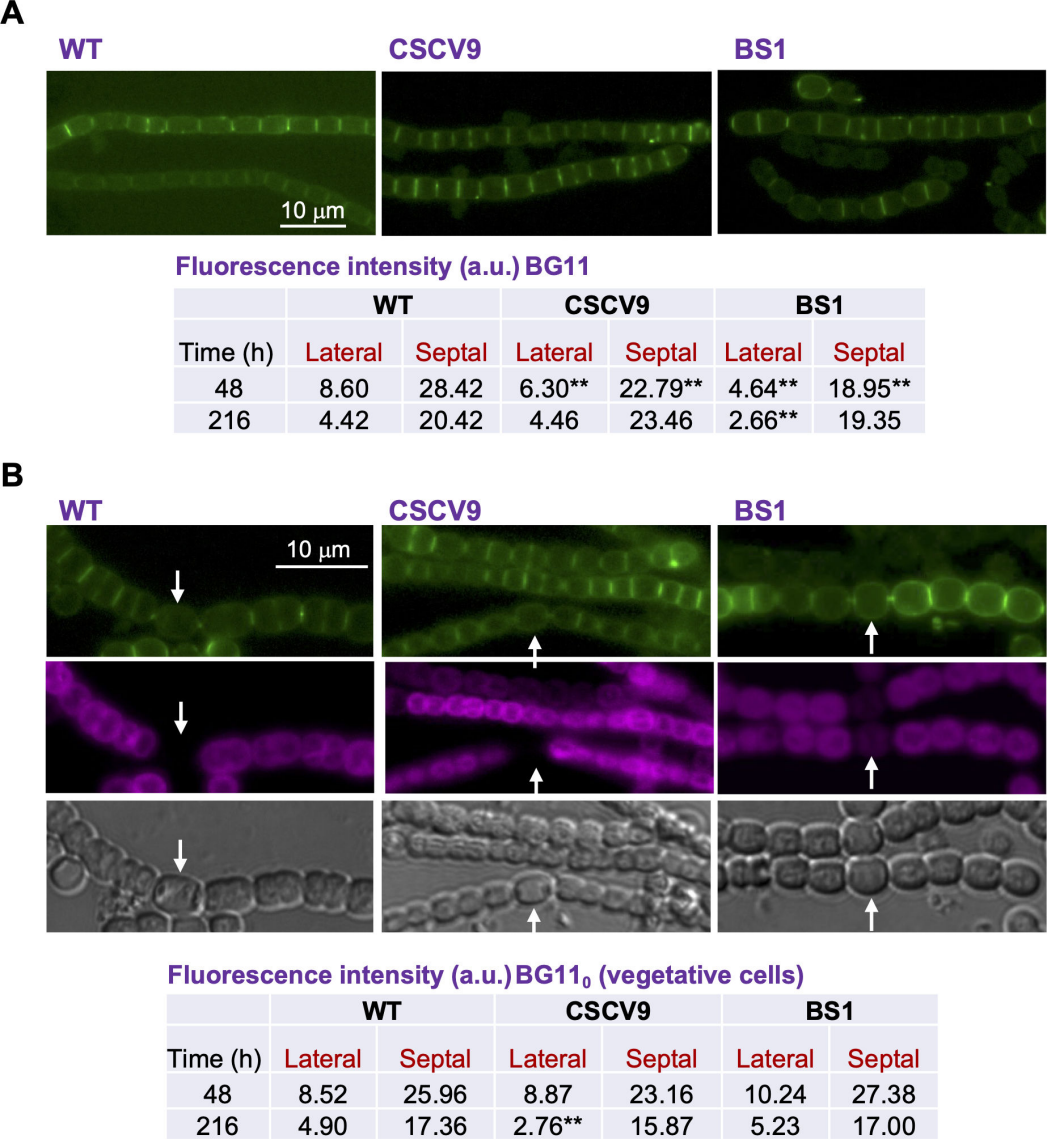
Localization of PG growth in *sepT* mutants. Strains *Anabaena* WT, CSCV9, and BS1 grown in BG11 medium were transferred (at a cell density of 0.5 µg Chl mL^−1^) to BG11 (**A**) or BG11_0_ (**B**) medium and incubated under culture conditions. After 48 hours, samples of filaments were stained with Van-FL and observed under a fluorescence microscope and photographed. Van-FL fluorescence (green), cyanobacterial autofluorescence (magenta), and bright-field images are shown. White arrows point to heterocysts. Magnification is the same for all micrographs. After 48 and 216 hours, lateral and septal fluorescence were quantified as described in Materials and Methods. Student’s *t*-test was used to assess significance of differences (Data Set S3). Significant differences (*P* < 0.01) are indicated by **.

The interaction of SepT with some proteins involved in lateral or septal PG growth, as suggested by the BACTH assays, led us to check whether the pattern of PG growth was affected by the mutation of *sepT*. For that, we used labeling with fluorescent vancomycin (Van-FL), which highlights the sites of PG incorporation, as done before in *Anabaena* [(22); see also reference (32)]. Filaments of *Anabaena* WT and strains CSCV9 and BS1 were incubated in BG11 or BG11_0_ for 48 (exponential growth) and 216 hours (slow growth) under the same conditions used for the determination of growth rates and labeled with Van-FL. In *Anabaena* WT, cells from BG11 cultures, as well as vegetative cells from BG11_0_ medium, presented weak peripheral and strong septal labeling with alternating intensities (lower in the more recently formed septa than the older ones), as well as labeling in the septum under construction during cell division (Fig. 6), as previously described [see references (32, 33)]. Filaments of CSCV9 and BS1 incubated in BG11 or BG11_0_ medium showed a labeling pattern similar to that of the WT (Fig. 6A and B). However, quantification of Van-FL fluorescence intensity in the cell periphery (lateral fluorescence) and in the septal regions indicates that after 48 hours in BG11 medium, both lateral and septal fluorescence were lower in the mutants than in the WT (Student’s *t*-tests *P* < 0.01), becoming more similar after 216 hours [at 216 hours only lateral fluorescence in BS1 appeared significantly lower (*P* < 0.01) than in the WT] (see the Table in Fig. 6A). In BG11_0_ medium, fluorescence intensity in vegetative cells appeared similar in the mutants and the WT [only lateral fluorescence at 216 hours was significantly lower (*P* < 0.01) in CSCV9 than in the WT] (see the Table in Fig. 6B).

### Localization of MreB, MreC, and MreD in strain CSCV9

**Fig 7:**
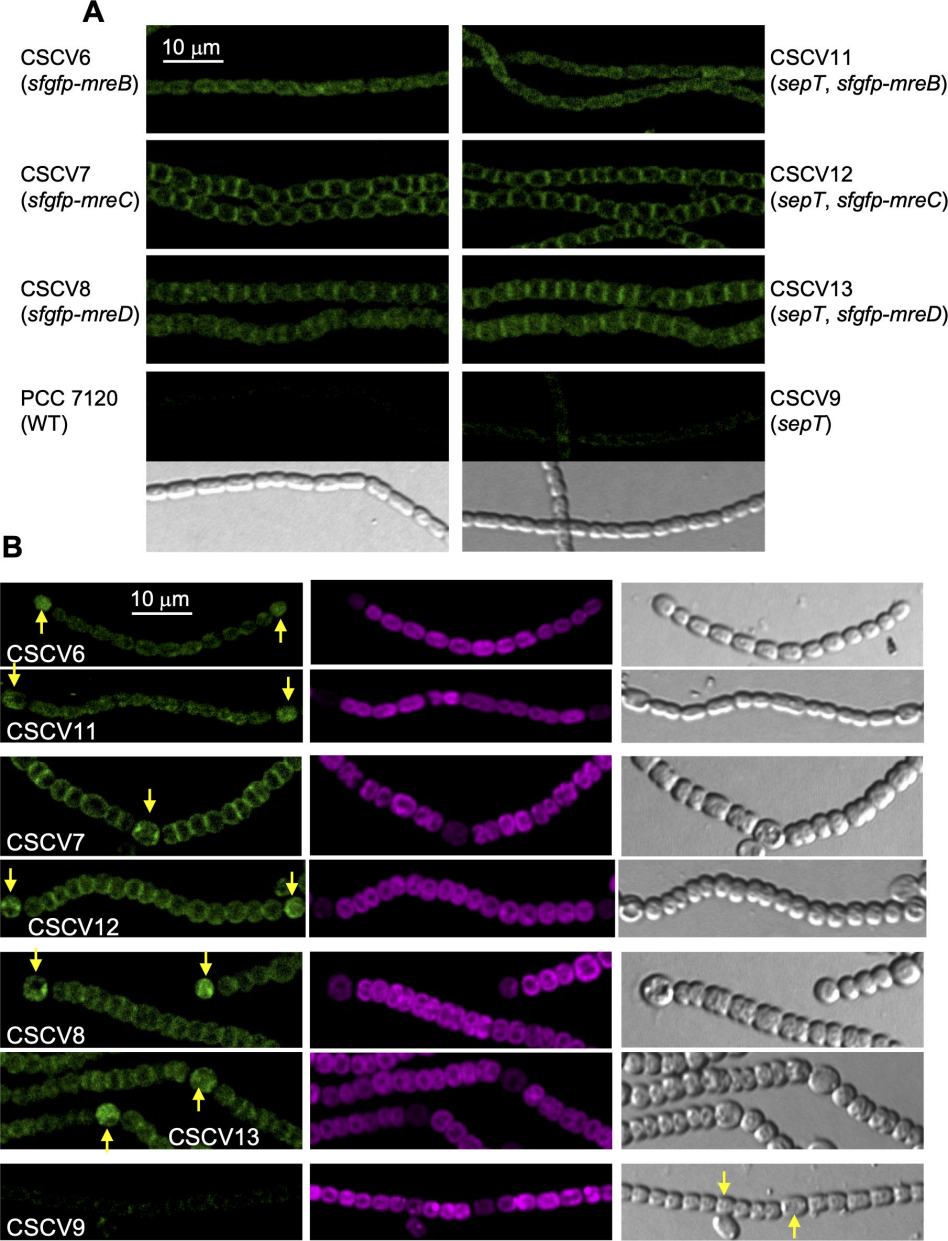
Localization of MreB, MreC, and MreD in the absence of SepT. Strains CSCV11 (*sepT*::C.K1, *sfgfp-mreB*), CSCV12 (*sepT*::C.K1, *sfgfp-mreC*), CSCV13 (*sepT*::C.K1, *sfgfp-mreD*), and reference strains *Anabaena* WT, CSCV9 (*sepT*::C.K1), CSCV6 (*sfgfp-mreB*), CSCV7 (*sfgfp-mreC*), and CSCV8 (*sfgfp-mreD*) were grown in BG11 medium and transferred to BG11_0_ + NH_4_^+^ (**A**) or BG11_0_ medium (**B**), adjusted to a cell density corresponding to 0.5 µg Chl mL^−1^. After 24 hours, aliquots of filaments were observed under a TCS confocal microscope and photographed. GFP fluorescence (green), cyanobacterial autofluorescence (magenta), and bright-field images are shown. Yellow arrows point to heterocysts. Magnification is the same for all micrographs.

We also studied whether inactivation of *sepT* affected the localization of the elongasome components MreB, MreC, and MreD. For that, we transferred gene constructs, whereby the *mreBCD* promoter region directs the expression of the fusion proteins sfGFP-MreB, sfGFP-MreC, or sfGFP-MreD to strain CSCV9, generating strains CSCV11, CSCV12, and CSCV13, respectively. Strains CSCV6, CSCV7, and CSCV8 express the same fusion proteins, respectively, in the WT background (32). The six reporter strains include, in addition, the intact *mreBCD* operon in its native genomic locus. In the presence of combined nitrogen, strains CSCV11, CSCV12, and CSCV13 exhibited GFP fluorescence localized through the cell periphery, including the intercellular septal regions, and at midcell, matching the divisome localization in dividing cells (Fig. 7A), a distribution similar to that described for strains, CSCV6, CSCV7, and CSCV8, respectively (32; Fig. 7A). Upon N-stepdown, vegetative cells of CSCV11, CSCV12, and CSCV13 exhibited peripheric, septal, and midcell GFP fluorescence similar to the pattern found in filaments incubated with nitrate. Heterocysts showed peripheric fluorescence and, frequently, fluorescence spots focalized at the cell poles (Fig. 7B). These observations also match the distribution observed in CSCV6, CSCV7, and CSCV8, respectively (Fig. 7B; 33), indicating that localization of MreB, MreC, and MreD is not noticeably affected in the *sepT* mutant background.

### Morphology of the septal nanopore structures in *sepT* mutants

**Fig 8:**
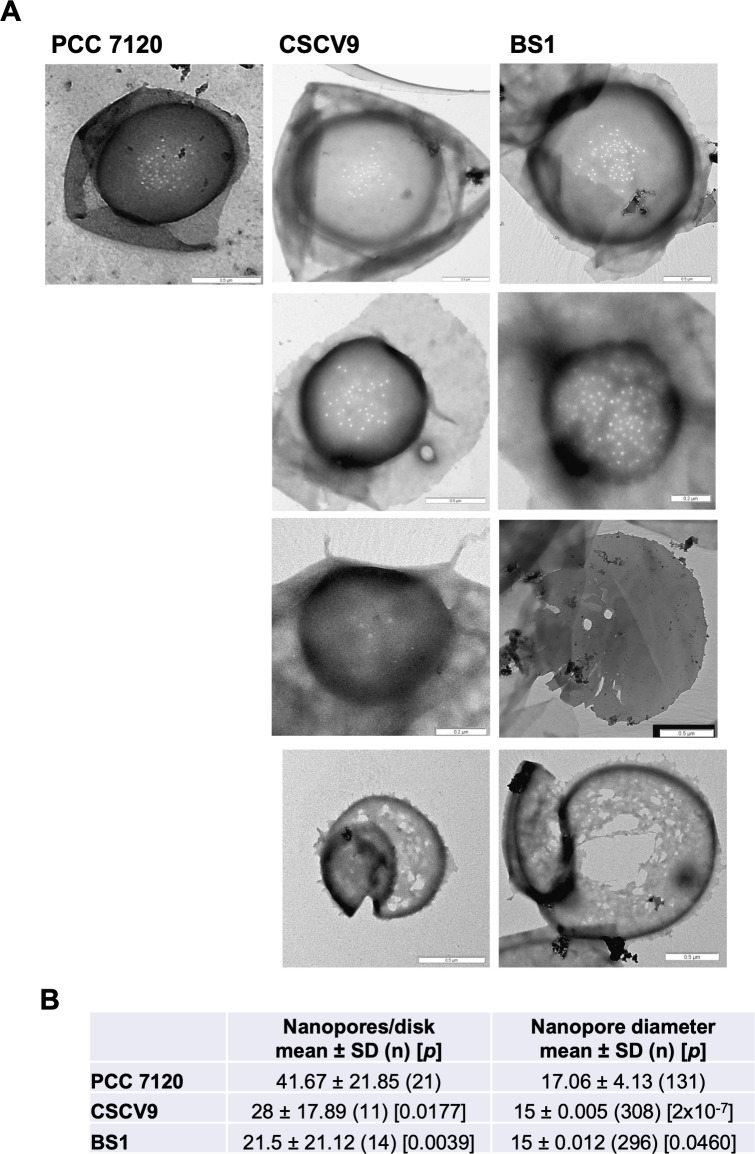
Septal nanopore array in *sepT* mutant strains. (**A**) Representative transmission electron microscopy images of PG sacculi septa from *Anabaena* WT and the *sepT* mutant strains CSCV9 and BS1 grown in BG11 medium (see Materials and Methods for details). Disks showing abnormal nanopore distribution (second line), few, larger than average, nanopores (third line), or nanopores severely enlarged and of abnormal shape (fourth line) are shown. (**B**) Number of nanopores per septum and mean nanopore diameter (nm). *n*, sample size (number of septal disks or nanopores, respectively). The difference between each mutant and the wild type was assessed by the Student’s *t*-test (*P* values are indicated). For comparisons between the two mutant strains, *P* values were 0.4933 for nanopore number and 0.5872 for nanopore diameter. Data on *Anabaena* WT are from reference (13).

Given that alterations in the number and morphology of septal nanopores have been described in mutants of other septal proteins in *Anabaena* (e.g., 13), we isolated PG sacculi from the *sepT* mutant strains CSCV9 and BS1 and observed the septal disks by TEM (Fig. 8; Fig. S3). In comparison to the WT, we observed a heterogeneity in the array of septal disk nanopores in the mutants. Septal disks in the WT contain an average of ca. 40 nanopores of about 17 nm in diameter concentrated in the central part of the disk (13; see Fig. 8). In comparison to the WT, septal disks of both CSCV9 and BS1 generally contained fewer nanopores with a slightly smaller size (Fig. 8B). However, although in some cases the mutant disks showed a nanopore distribution that resembles the WT pattern (first line in Fig. 8A), disks with nanopores distributed throughout the whole disk area (second line in Fig. 8A) or including only two to four, larger than average, nanopores (third line in Fig. 8A) were also observed. In addition, some disks showing pores severely enlarged and of abnormal (non-circular) shape, apparently resulting from PG mesh breakage, were also frequently detected in both mutants (fourth line in Fig. 8A). Figure S3 shows that peptidoglycan isolated from filaments of BS1 grown on agar plates presented a distribution of septa similar to that found in liquid medium.

## DISCUSSION

In this work, we have identified the product of *Anabaena* ORF *all2460*, a cyanobacterial cytoskeletal protein linked to the divisome, the elongasome and the septal-junctions, which we have termed SepT. SepT is predicted to be a coiled-coil-rich protein characteristic of filamentous cyanobacteria capable of heterocyst differentiation. Indeed, homologs to SepT have been found encoded in the genomes of all tested heterocyst-forming strains. As in most cases, *Anabaena* SepT is predicted to be an inner membrane-anchored cytoplasmic protein and as most CCRPs, it appears able of self-interactions (Fig. 3B), with predicted formation of structured dimers (Fig. 1C).

In *Anabaena*, SepT is localized in the cell periphery and the intercellular septa, and in dividing cells, it is also detected at midcell matching the localization of the divisome (Fig. 2). Localization at the cell periphery and the divisome has also been described for the elongasome components MreB, MreC, and MreD in *Anabaena* (32). Moreover, we have detected SepT interactions with MreB (Fig. 3B), invoking a role of SepT in the elongasome function. We have not detected any apparent alteration of MreB, MreC, or MreD localization in the absence of a functional SepT protein (Fig. 7). However, two different *sepT* mutants showed impaired regulation of cell size (Fig. 5B), which might reflect a discoordination between cell growth and division, and altered PG growth with lower incorporation, especially under conditions of higher growth rate (exponential growth with combined nitrogen) in the mutants (Fig. 6A). In addition, we have detected interactions of SepT with putative RodA (Fig. 3A), the elongasome glycosyl-transferase catalyzing PG polymerization, as well as with three putative PBPs (All2981, All2952, and Alr0718). These results suggest a role for SepT in the regulation of PG expansion during cell growth.

The localization of SepT in the intercellular septa and the divisome coincides with that reported for other septal proteins that play important roles in filament maintenance and intercellular communication in *Anabaena*, including SepJ, FraC, and SepI. Thus, SepT could have a function related to the specialized structure of the intercellular septa of heterocyst-forming cyanobacteria. Notably, our past and current investigations suggest that interaction with the divisome during cell division is a mechanism for the localization of proteins that remain in the intercellular septa once the division event has concluded. Indeed, SepJ has been shown to interact with the divisome components FtsQ and ZipN (40, 43), and also with SepI, which interacts with FtsI, SepF, and ZipN (20). Here, we have detected clear interactions of SepT with ZipN, SepF, and FtsW. This, together with its localization to the divisome, points to a similar mechanism for SepT localization to the intercellular septa.

The interaction of SepT with putative FtsW (divisome PG glycosyl-transferase) suggests an effect on septal PG growth or remodeling. Indeed, we have observed conspicuous alteration on the nanopore array of the septal PG disks in two different *sepT* mutants, including a lower average nanopore number, altered nanopore distribution, and even very aberrant septal disks with only two to four large nanopores or showing apparent tears in the PG mesh (Fig. 8). SepJ, with which SepT also interacts (Fig. 3B), is also a coiled-coil-containing protein that influences the septal disk nanopore array in *Anabaena*, although *sepJ* inactivation had milder effects than *sepT* inactivation leading to reduced nanopore numbers (15). In conclusion, besides influencing lateral PG growth, SepT is a new component of the specialized septal structure of filamentous heterocyst-forming cyanobacteria directly linked to the cell growth and cell division machineries. It has a role in the processing of the intercellular PG required for the formation of intercellular communication structures that are key feature of multicellular organisms.

## MATERIALS AND METHODS

### Bacterial strains and growth conditions

*Anabaena* sp. PCC 7120 and mutant strains were grown photoautotrophically in BG11 medium (containing NaNO_3_) or in BG11_0_ (lacking combined nitrogen) (6), or in BG11_0_ supplemented with 4 mM NH_4_Cl and 8 mM TES-NaOH buffer, pH 7.5 at constant illumination of 12–30 photons m^−2^ s^−1^ intensity. Cells were either grown in Erlenmeyer flasks with shaking or on medium solidified with 1% (wt/vol) Difco agar. For the mutants, media were supplemented with antibiotics: spectinomycin (Sp) and streptomycin (Sm) at 5 µg mL^−1^ each in solid media or at 2 µg mL^−1^ each in liquid media, or with neomycin (Nm) at 25 µg mL^−1^ in solid media or 5 µg mL^−1^ in liquid media. Chlorophyll content of the cultures was determined after extraction with methanol (53). In *Anabaena*, 1 µg chlorophyll corresponds to ca. 3.3 × 10^6^ cells (54). (Table S1 and S2 list all used cyanobacterial strains, plasmids, and oligonucleotides.)

### Distribution of homologs in cyanobacteria

Homologs to SepT were detected in completely sequenced genomes publicly available in RefSeq (version 01/2021) by amino acid sequence similarity using stand-alone BLAST (55, 56) (v. 2.2.26). Protein sequences that were found as BLAST hits with a threshold of *E*-value ≤1×10^−5^ were further compared to SepT by global alignment using needle (57). Hits having ≥30% identical amino acids in the global alignment were considered as homologs. For the phylogenetic analysis, homologs in a set of representative heterocystous species were manually selected and complemented by all homologs in non-heterocystous species. Amino acid sequences were aligned with MAFFT (v. 7.475) (58). The phylogenetic tree was inferred with IQ-TREE (59) (v. 1.6.12) with restricted automatic model selection to the Le & Gascuel model (60) and fast bootstrap with 1,000 replicates. The tree was rooted using the MAD approach (61). The phylogenetic tree was visualized with FigTree (http://tree.bio.ed.ac.uk/software/figtree/).

### Computational prediction

Coiled-coil-rich regions were predicted using COILS (v. 2.2) (47), and conserved protein domains were predicted using NCBI Conserved Domains Search (62). Prediction of subcellular localization was done using TMHMM (v. 2.0) (63), PSORTb (v. 3.0.2) (64), Gneg-mPLoc (v. 2.0) (65), PSIPRED (v. 4.0) (66), and Phobius (67). The employed putative promoter site for *sepT* (1,470 bp upstream of the ORF) was based on promoter and transcription factor binding site predictions by BPROM (68).

### Plasmid and *Anabaena* mutant construction

Mutant strain CSCV9 carries a version of the *sepT* gene in which codons 69–367 have been substituted by gene-cassette C.K1 encoding Km/Nm resistance. To generate it, two DNA fragments were amplified from *Anabaena* genomic DNA using the primer pairs all2459-1/all2460-2 (encompassing sequences internal and upstream of *sepT*) and all2460-1/all2461-1 (encompassing sequences internal and downstream of *sepT*), including terminal restriction sites BamHI and PstI. Both fragments were joined together by overlapping PCR, and the resulting single fragment was cloned into mobilizable vector pRL277 [encoding the gene *sacB* for positive selection (69)]. Gene-cassette C.K1 from plasmid pRL161 (70) was then inserted into the internal BamHI site, generating plasmid pCSCV36, which was transferred to *Anabaena* by conjugation (71).

Mutant BS1 carries gene cassette C.S3, encoding Sm/Sp resistance, substituting for the *sepT* gene. To generate it, 1,500 bp upstream and downstream of the *sepT* ORF were amplified by PCR from *Anabaena* genomic DNA using primers 842KO_2A/842KO_2B and 842KO_4A/842KO_4B, respectively. The upstream and downstream *sepT* regions flanking the C.S3 cassette [amplified with primers CS.3_Fwd/CS.3_Rev using pCSEL24 (72) as a template] were then inserted into PCR-amplified pRL278, including the gene *sacB* for positive selection (73), using primers pRL271_Fwd/pRL271_Rev by Gibson assembly, yielding pTHS109, which was transferred to *Anabaena* by conjugation. For both CSCV9 and BS1 mutants, insertion of the mutagenic construct by double crossover was selected by resistance to sucrose and lack of antibiotic resistance encoded in the vector portion of the transferred plasmid. Both mutants were segregated for the mutant chromosomes, as tested by PCR analysis (not shown).

For the generation of strains CSCV11, CSCV12, and CSCV13, strains expressing a *sfgfp-mreB, sfgfp-mreC,* or *sfgfp-mreD* fusion gene, respectively, expressed from the native *mreBCD* operon promoter (P*_mreB_*), strains CSCV6, CSCV7, and CSCV8, which, respectively, express the same fusion genes in the WT background, were used as recipient of plasmid pCSCV36. Insertion of the mutagenic construct and segregation for the mutant chromosomes were verified by PCR analysis as above.

The replicative plasmid pTHS240, used for the localization of SepT-GFP, included a *sepT-gfpmut3.1* fusion gene under the control of the native promoter (predicted by BPROM). To generate it, the *sepT* promoter sequence and ORF were amplified using the primer pair p842_25C_long_A/Nos842_2B; the *gfpmut3.1* sequence was amplified using the primer pair GFP_842_A/GFP_25C_R, and both PCR products were cloned into PCR-amplified plasmid pRL25C (74) using primer pair pRL25C_F/pRL25C_R by Gibson assembly.

The replicative plasmid pTHS143, driving the expression of SepT-GFP from P_petE_ used for co-IP analysis, was cloned as follows: the *petE* promoter was amplified using primer pair petE_903_Fwd/ pRL25c_NEB_Rev; *sepT* was amplified using primer pair Nos842_2 A/ Nos842_2B, and *gfpmut3.1* was amplified using pRL25c_NEB_Fwd/ pRL25c_NEB_Rev, and all three PCR products were cloned into PCR-amplified pRL25C using primer pair pRL25c-903_V_F/ pRL25c-903_V_R.

### BACTH analysis

BACTH assays were based on the reconstitution of adenylate cyclase from *Bordetella pertussis* (75). Genes were amplified by PCR using *Anabaena* DNA as template and oligonucleotide pairs: MB_25 A/MB_25B (SepT-T25), MB_26 A/MB_26B (T25-SepT), MB_27 A/MB_27B (SepT-T18), MB_28 A/MB_28B (T18-SepT), alr0653-1/alr0653-2 (T25-RodA), alr0653-3/alr0653-2 (T18-RodA), alr0653-3/alr0653-4 (RodA-T25, RodA-T18), alr5045-7/alr5045-8 (T25-Alr5045), alr5045-9/alr5045-8 (T18-Alr5045). The resulting PCR products were cloned in pUT18, pUT18C, pKNT25, or pKT25. All the resulting plasmids were verified by sequencing. Other fusions used in this work were as previously described (20, 32, 36, 40, 43). Plasmids were transformed into *Escherichia coli* XL1-Blue for amplification. Isolated plasmids were co-transformed into strain BTH101 (*cya*-99), and the transformants were plated on solid LB medium containing selective antibiotics and 1% (wt/vol) glucose.

β-galactosidase activity was measured after growth in liquid medium in the presence of IPTG and antibiotics, using *ο*-nitrophenol-β-galactoside as a substrate. Either the *ο*-nitrophenol produced per milligram of protein versus time was represented and the β-galactosidase activity deduced from the slope of the linear function, or the *ο*-nitrophenol produced was recorded in Miller units as described in the manufacturer’s manual (Euromedex).

### Co-immunoprecipitation

About 20–30 mL of culture of *Anabaena* WT or a derivative strain including plasmid pTHS143, driving the expression of SepT-GFP from P_petE_ promoter, was pelleted by centrifugation (4,800 × *g*, 10 minutes, RT); cells were washed twice by centrifugation (4,800 × *g*, 10 minutes, RT) with 40 mL PBS and then resuspended in 1 mL lysis buffer [PBS-N: PBS supplemented with 1% (vol/vol) NP-40] supplemented with protease inhibitor cocktail (PIC; cOmplete, EDTA-free Protease Inhibitor Cocktail, Sigma-Aldrich). Cells were lysed using the VK05 Lysis Kit (Bertin) in a Precellys 24 homogenizer (3 strokes for 30 seconds at 6,500 rpm), and cell debris was pelleted by centrifugation (30 minutes, 21,100 × *g*, 4°C). Fifty microliters of μMACS Anti-GFP MicroBeads (Miltenyi Biotec) were added to the resulting cell-free supernatant with further incubation for 1 hour at 4°C with mild rotation. Afterward, the samples were loaded onto µColumns (Miltenyi Biotec), washed twice with 1 mL lysis buffer, and eluted in 50 µL elution buffer [50 mM Tris HCl pH 6.8, 50 mM DTT, 1% SDS, 1 mM EDTA, 0.005% (wt/vol) bromophenol blue, 10% (vol/vol) glycerol; Miltenyi Biotec]. Until further use, samples were stored at −80°C.

### Mass spectrometry analysis

Mass spectrometry was performed as previously described (20). The acquired MS/MS data were searched with the SequestHT algorithm against the entire reviewed Uniprot protein database of *Anabaena* sp. PCC 7120, including proteins encoded in plasmids (6,922 sequences in total). Static modification applied was carbamidomethylation on cysteine residues, while oxidation on methionine residues was set as dynamic modification. Spectra were searched with full-enzyme specificity. An MS mass tolerance of 10 ppm and an MS/MS tolerance of 0.02 Da were used. Proteins were identified with at least three unique peptides with a FDR confidence ≤0.01 (high).

### Van-FL staining and quantification

Filaments were stained by incubation with 2 µg mL^−1^ Vancomycin-FL (Bodipy-FL conjugate, Invitrogen), and lateral and septal fluorescence were quantified with ImageJ (76) processing of fluorescence images by collecting total fluorescence in manually defined equal square sections, as described (32). For each cell, lateral fluorescence was calculated as the mean of the values of four sections and septal fluorescence as the mean of two sections, one at each cell pole. Twenty to thirty cells were counted for each strain and condition, and the average values were calculated.

### PG sacculi isolation and visualization

PG was isolated from filaments grown in BG11 medium by protease treatment and hot detergent extraction, as described (15). Aliquots of the obtained preparations were deposited on formvar-carbon film-coated copper grids and stained with 1% (wt/vol) uranyl acetate and examined with a Zeiss Libra 120 plus (Zeiss) transmission electron microscope at 120 kV.

### Microscopy

Cell area and cell axis length were determined automatically by processing light-microscopy images with ImageJ software, as in reference (31). Data were plotted using the open-source software RStudio Desktop (https://www.rstudio.com). Van-FL fluorescence was visualized with a Leica DM6000B fluorescence microscope and a FITCL5 filter (excitation band-pass, 480/40; emission band-pass, 527/30) and photographed with an ORCA-ER camera (Hamamatsu). GFP fluorescence was monitored with an Olympus TCS SP2 confocal laser-scanning microscope equipped with an HCX PLAN-APO 63 × 1.4 NA oil immersion objective (excitation, 488 nm; collection, 500–540 nm for GFP or 630–700 nm for cyanobacterial autofluorescence) or with an Olympus FLUOVIEW FV3000 (hyper-resolution) confocal laser-scanning microscope equipped with an UPlanApo 60 × 1.5 NA oil immersion objective (excitation, 488 nm; collection 500–540 nm for GFP or excitation, 640 nm; and collection, 650–750 nm for cyanobacterial autofluorescence).

## SUPPLEMENTAL MATERIAL

Data Set S1 mbio.00983-23-s0001.xlsx Sequences used for SepT phylogenetic tree.

Data set S2 mbio.00983-23-s0002.xlsx Excerpt of proteins co-precipitated with SepT-GFP.

Data Set S3 mbio.00983-23-s0003.xlsx Statistics of lateral and septal Van-FL fluorescence intensity.

Fig. S1 mbio.00983-23-s0004.pdf Sequence alignement of SepT homologs.

Fig. S2 mbio.00983-23-s0005.pdf Localization of truncated SepT-GFP in Anabaena.

Fig. S3 mbio.00983-23-s0006.pdf Septal nanopore array in strain BS1 grown on solid medium.

Table S1 mbio.00983-23-s0007.pdf Cyanobacterial strains and plasmids used in this work.

Table S2 mbio.00983-23-s0008.pdf Oligodeoxynucleotide primers used in this work.

ASM does not own the copyrights to Supplemental Material that may be linked to, or accessed through, an article. The authors have granted ASM a non-exclusive, world-wide license to publish the Supplemental Material files. Please contact the corresponding author directly for reuse.
