# Supplementary material for: SepT, a novel protein specific to multicellular cyanobacteria, influences peptidoglycan growth and septal nanopore formation in Anabaena sp. PCC 7120
Source: mBio. 2023 Aug 31;14(5):e00983-23. doi: 10.1128/mbio.00983-23 (PMC10653889; doi:10.1128/mbio.00983-23)
Supplement: Fig. S1 — Sequence alignement of SepT homologs. [file mbio.00983-23-s0004.pdf]

SepT MULTIPLE SEQUENCE ALIGNMENT


```
                                                                                         :   .. .       :            ::   :          ::      .              . ::     *  :   :  ..   .
Nostoc_sp._PCC_7120_=_FACHB-418          ------------------MQQVIVSNRFILGIVAFSVSFGLSLVPK-WNFSQAFFTGVITVVATYAAALFVD----KRRRNYELLL-LSSLRKRIQETEGLKSRLAREI-------EQLETHRSLLYTESQQLKNQILDSRNQRDSLHRD   119
Nostoc_punctiforme_PCC_73102             ------------------MQSVTISNRFILGIVAFSVSFGLSLVPS-WDFNKAFLTGLITAATIYAAALFVD----KRRRNYEMFV-LGSLRKRIKEMEGLKARLVREI-------NQIEEHHNLLYAESQQLQNQVTESRNQRDSLHRE   119
Nostoc_azollae_0708                      MTLITTNSDKFTTLKTSNMQSAIVSNRLILCVFAFSVSFGLSLVPN-WDFTKALLTAIITVLATYSAALFVD----KRRRNYEMLV-LSSLHRRIRELEGLKYRIAREI-------KQVEEHKTILYTESQQLQNQIAECRNQRDSLHRE   137
Fischerella_thermalis                    ------------MLKTSNMQSAIVSNRLILGVVAFGASFGLGLVFS-WDLKNAFLTGIITIFSTYLAALFID----KRRRASEMLV-LDSLHRRIKELEGLKSRIIAEV-------NQLESHHSLLYQESNKLQNQIVERRNQRDSLNRE   125
Tolypothrix_sp._PCC_7601                 ------------------MHSAILSNRLFLGIVAFSVSFGLSLVPN-WDYSKAFVTGIITVIATYAAALSVD----RRRKNHEMLI-LSSLRKRIKELEELKSRIAREV-------HQIEEHRNSLYTESKNLQNKVAESRNQRDSIQRE   119
Spirulina_subsalsa_PCC_9445              M----------------PSRNKKLVNQVILGSIAFSLSFGLGLLLS-REFKQGVLTGIAALPASYGCAIALY----RKRVQQDKQL-KQGLHHQIQLLLQQRDNCQEEIAIALHQRQTLESNLDALYSEREQILMRVAELHQYRKELYQS   128
Mastigocladopsis_repens_PCC_10914        ------------MLKTSNMQSAMVSNRFIIGIIAFGVSFGLSLVLS-WDVSKAFVTGVITVPATYLSALFVD----KRRRNHEMLI-LDSLQKRIRELEGLKSRFVAET-------NQIEANNALLYAESNKLQNQVLERRNQKETLSRE   125
Rivularia_sp._PCC_7116                   ------------MLKTSNMQDSMVSNRFIIGIVAFGVSFGLTLVFTWWNFYTAIVTGIITILATYTATFIVDKKAEKKRRHYELQV-VDSYYKRIQELESVQHGITLEV-------DRLEATRRALHQESNQLQHQVGDRRNQRDSLNRE   130
Chlorogloeopsis_fritschii_PCC_9212       ------------------MQSAMASNRFILGIVAFGISFGLSLALT-WNFGKSLIIGLITLIVTY-SALFVE----QQQRNHEMLARRDSLHRRIRELEGLKSRMLAEI-------NQLEAHHALLYQESNKFQNQVVERRNQRDILNRE   119
Chlorogloeopsis_fritschii_PCC_6912       ------------------MQSAMASNRFILGIVAFGISFGLSLALT-WNFGKSLIIGLITLIVTY-SALFVE----QQQRNHEMLARRDSLHRRIRELEGLKSRMLAEI-------NQLEAHHALLYQESNKFQNQVVERRNQRDILNRE   119
Microcoleus_sp._PCC_7113                 ------------------MQSIRVVNRILIGSVAFSVSFLLGLLVN-RDINKALLTGVITVPGTYAGAAIAE----KRRIYQEKLL-RGSLQNQIQELLEEENQLYQSLASATETRQEVEASINALQGERSQLLNRVSDLHIQRNELYRE   126
Gloeocapsa_sp._PCC_7428                  ----------------------MVSNRIILGAVAFGVSFGISFLSN-RNANRALLTSLITLPATYAAAIVVD----KRQNNRELLV-LSSQRQKIYELEAQEINLNRILSNANFKKQELENSIHYLQTECNQLRSQFEARHHYKDDLQQE   122
Nodularia_spumigena_CCY9414              ------------------MQSAMISNRYILGIVAFSVSFGISLVPN-WDLNQALITGVITILATYAAALFTD----KRRSKYEMLI-LTSHRKRIKEMEGLKLRIVKEI-------NQIEEHRKLLYAESQKLEKQILDCRNQRDSLHRD   119
Scytonema_hofmannii_PCC_7110             ------------MLKISSNQSTMNSNRFIIGIIAFSVSFSLSLFLS-WDFNKAFLTGLVTVPATYLAAFFVD----KRRRNNEMLV-LDSLQRRIQELEGLKFRIVSDI-------KQLEAHNALLYSESSKLQNQLVERRTQRDSLHRE   125
Richelia_intracellularis_HH01            ------------------MQSAMLSHRFVLGIIALGVSFGLSLALT-WDFRDALITGIITITITYTL-LFIE----HKQNRYEMKGKIDLLQRRVKELEVLKKHIIGEI-------NQLEAYSSSLQMESNKLKNQIE--KNQ-----RE   112
Aphanizomenon_flos-aquae_NIES-81         MTLITNNSNNLATIKPSKMQPTIVSHRVVLGITAFSVSFGLSLVPN-WDISRAFLTGIITVLATYTAAFFVD----KRRRNDEWKM-IGSLQRRIRDIEGLKSRILKEI-------HQLEEYRNLLYAETQQLENQIGDCRSQRDSLHRD   137
Aphanizomenon_flos-aquae_2012/KM1/D3     MTLITNNSNNLATIKPSKMQPTIVSHRVVLGITAFSVSFGLSLVPN-WDISRAFFTGIITVLATYAAAFLVD----KRRRNDEWKM-IGSLQRRIRDIEGLKSRILKEI-------HQLEEYRNLLYAETQQLENQIGDCRSQRDSLHRD   137
Spirulina_major_PCC_6313                 --------------------------------------------MH-QQLAPALITAVIMVPVGYFVSLWSE----FRHLQDDRHQ-RNSLRHQIHTLQAQHHNLQQTIQATLQTQQEVNGTLQRLELDRDRLLSRITELHQYRNELIHE   100
Nodularia_sp._NIES-3585                  ------------------MQSAMISNRYILGIVAFSVSFGISLVPN-WDLNQSLITGVITILATYAAALFID----KRRSNYEMLI-LSSHRKRIKEMEGLKLRIVKEI-------NQIEEHRNLLYAESQKLEKQILDCRNQRNILHRD   119
Anabaenopsis_circularis_NIES-21          ------------------MQAVMVSNRFLLGIVAFSVSFGLSLVPN-WNFSQAFITGIITVLATYAAALFVD----KRRRHHEMLV-LVTLRKRIKEQEALKNRIYREI-------KQIEEHRSLLYAENQKLQNQIAESRNQRDSLHRE   119
Calothrix_sp._NIES-2098                  ------------------MHSATVSNRLFLGIVAFSVSFGLSLVPN-WDYTKAFVTGIITLFATYAAAFCVD----KRRKNHEMLV-LNSLRKRIRELEELKSRIVREI-------DQIEEHRSSLYAESKQLQNKVTECRNQRDSLHRE   119
Chroococcidiopsis_sp._TS-821             ----------------------MVNNRIVLGAVAFGVSFGISFLAN-RNANRALLTGLITLPATYAAAIVVD----RRQKHRELLV-LASQRQKIHELETQEVNMNRILSNANLKKQELENSINYLQTECHQLRSQFEVRHQYKDDLEKE   122
Calothrix_desertica_PCC_7102             ------------------MQPAVSSNRFLIGTIAFVLSFGLSLIFK-WNFGEAVFTGIITVMATYIAAFCVD----KRRRNYEMLI-LESYRKRIRELEGVEANFIAEI-------NQLENHRNILYNETNQLQSQISERRNQREILNRE   119
Cylindrospermum_sp._NIES-4074            MTLKTTYSENLTRLKTSNMQSVIISNRLFLGIVAFSVSFGLSLVPN-WDFTKAFFTGVITVIATYVAAFCVD----QRRKNHEMLV-LTSLRKRIKELEGLKSRIVREI-------NQIEEHRSILYAESSQLQNQIAERRNQRDIFHHD   137
Pleurocapsales_cyanobacterium_LEGE_06147 ------------------MESPKVINKVLLSSVAFGVSFGIGMLVSEKDIKNALTTGALAATAGFAGALVSE----RKKADGENLV-EDSLQTQVQELKTQEDWLHQSIAEATTKMQEVEAGINSLQTEHAQLLENIADLN---------   118
Anabaenopsis_elenkinii_CCIBt3563         ------------------MQSAIISNRYILGIIAFSVSFGISLVPQ-WNFNQALITGIITIISTYAATLFID----RRRSNYEMMI-LNSNRQRIKELEGLKRRLVKEV-------NQIEEIRNLLYADSQSLENQILESRTQRDKVHRD   119
                                         1.......10........20........30........40........50........60........70........80........90.......100.......110.......120.......130.......140.......150
```

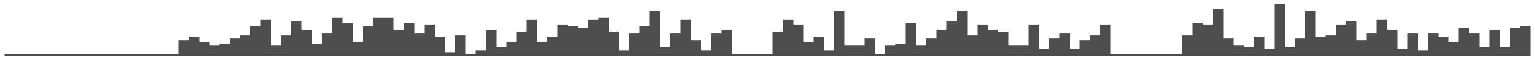

```
                                                                                                                        .                             .    :   .                     .   :  :
Nostoc_sp._PCC_7120_=_FACHB-418          LGIFASQR-----------KQIESELANLKSTICSLE-----------------------------------ENQIELNNSLSTLASEKRRLESNHHVC----------RAEITQLRNQIS----DLNQE-------KQELENSITLLGR   202
Nostoc_punctiforme_PCC_73102             LRTFAGQK-----------KQLETEINSLQTEINNLQ-----------------------------------KNQTELNNAFSILTAEKRRLESNCNVS----------RAEITQLQSQIS----ELQQE-------RQEVESNLTLLGR   202
Nostoc_azollae_0708                      LGTFAGQK-----------KQLELEINNFTTELHKLQ-----------------------------------NNTEKLNNSCSELAAEKRRLELNCNVF----------RSEITQLKAQIE----AFKQE-------KQEIENNLVLINR   220
Fischerella_thermalis                    LSSCVIEK-----------KQLEAQISQLHNEIQELE-----------------------------------KAKLEHNNSLSAINTEKRRLELSCSVS----------KAEINQLNNQLC----ELLQQ-------RQDLESNITLLER   208
Tolypothrix_sp._PCC_7601                 LSTFAGQK-----------KQFEHEITVISTEIHNLE-----------------------------------QNKTELNNSVSMLNAEKRRLELNCNVS----------RSEINQLQHQIS----ELLQE-------KEEIENNLTLLGR   202
Spirulina_subsalsa_PCC_9445              LPT--QQQ-----------KTLLGTNPWQSPQLQAAP-----------------------------------DHPVEFKRGGVAHGVLVQKSEDRLEQL----------QSEYDQLQGQIL----AQQTK-------REQLIPELTQLSR   209
Mastigocladopsis_repens_PCC_10914        LSNYVIEK-----------KHLESEINYLQNEFHKLE-----------------------------------KSKVELNNSLSALTVDKRRLELNYNVS----------RSEITQLQTQIS----ELQQQ-------KEELESNLTLLNR   208
Rivularia_sp._PCC_7116                   LSTFALQK-----------RHLESEVVNLRDELKNLE-----------------------------------KSKQEAENSFANITAEKRRLELNCNVS----------KSEITQLHAKIG----ELLQQ-------REELDSNLILLDR   213
Chlorogloeopsis_fritschii_PCC_9212       LSSFIIEK-----------KQLESQINYLQDEISTLE-----------------------------------STKTEINKSFSNLNAEKRRLELNYNVS----------KAEINQLQTQIS----QLQQQ-------KQELESNLVLLGR   202
Chlorogloeopsis_fritschii_PCC_6912       LSSFIIEK-----------KQLESQINYLQDEISTLE-----------------------------------STKTEINKSFSNLNAEKRRLELNYNVS----------KAEINQLQTQIS----QLQQQ-------KQELESNLVLLGR   202
Microcoleus_sp._PCC_7113                 LSDFQKQK-----------QQQETEFYHLQTQIQRLE-----------------------------------RQQVELEQSLSAKAFQLNQTESRANQL----------KKEIERLHKQIS----YKKQQ-------KEQLHPDLITLEN   209
Gloeocapsa_sp._PCC_7428                  LKILKEQK-----------EDLEKELNSLHANLCELEHQKATVFTEKKALEQNSDALRLELAQLHRDFTVTTQQKEALEQEIANLHQQQSALSSQLKSLHIQINSLKTQHNQENQAIQELRLEQDKLQKQLIQQQSEKVALEVDLQSFKE   261
Nodularia_spumigena_CCY9414              LGIFAGQK-----------KQLETESINLTAEIATLE-----------------------------------QNQAELHHAFSQLTTEKRRLELNCNTS----------RAEIMQLQNKIG----ELQQE-------KQELESNVSLLGR   202
Scytonema_hofmannii_PCC_7110             LSSFFIEK-----------QQLESEIHYLQNQLHTLD-----------------------------------QTKTDLNHSFSALNTEKRRLELNCNVS----------RGEIAQLQNQLL----ELQQQ-------KQELESSITLIYR   208
Richelia_intracellularis_HH01            FSSLGVEK-----------KAIERQINQLTLEVNNLQ-----------------------------------ANIDQSSNYLDELNKEKRSLDINGNLA----------KAEITQLKSQID----ELQEQ-------KQDLENNLTLLNR   195
Aphanizomenon_flos-aquae_NIES-81         IGTFAAQK-----------KQIEAEIYNLKNEFVHLE-----------------------------------NSNIELNNSCNDLIAEKRRLELNCHAS----------RVEINQIQPQID----TIKQQ-------KKELENDVILLER   220
Aphanizomenon_flos-aquae_2012/KM1/D3     IGTFAAQK-----------KQIEAEIYNLKNEFAHLE-----------------------------------NSNIELNNSCNNLIAEKRRLELNCHAS----------RAEINQIQPQID----TIKQQ-------KKELENDVILLER   220
Spirulina_major_PCC_6313                 LHQYQQAQTPNPYPQSLDIATATATQAQQQNQAQQLE-----------------------------------QYLHRLNEAIEAAQTDILDQQEALEAV----------QQAVTQLTAEKA----TLQSQ-------TSQLHQTLGSLKA   194
Nodularia_sp._NIES-3585                  LGTFAGQK-----------KQLETDSINLTAEIATLE-----------------------------------QNQAELHHAFSQLTTEKRRLDLNCNTS----------RAEILQLQNQIS----ELQQE-------KQELESNVSLLGR   202
Anabaenopsis_circularis_NIES-21          LSNFAGQK-----------KQLETETNNLKNTIKTLE-----------------------------------KTSTELSNTCSNLTAEKRRLELHSNVS----------HAEIVQLQTQIA----QLRQD-------KQELENNLTLLGR   202
Calothrix_sp._NIES-2098                  LSTFAGQK-----------KQLENELSVLKAEVHSLD-----------------------------------KNKAELNNSFSVLTSEKRRLELNCNVS----------RSEINQLQNQIS----ELQQA-------KQELESNLTLLGR   202
Chroococcidiopsis_sp._TS-821             LEILK-------------------------TSLRELEQEKATLFAEKKVLEE--------------DFTTKTRSKEALEQEISNLHQQKSTLLSQLESL----------NSQYNQIVKKIQIEQIKFTHQ-------KSELEADLQTFKE   216
Calothrix_desertica_PCC_7102             LGSFVGQK-----------AALETTYNRLQHEIENIE-----------------------------------RSKIELHNSFA---AEKRKQDLNVSVC----------RAEITQLQIHIA----ELKKQ-------KEEFENNLTLLER   199
Cylindrospermum_sp._NIES-4074            LSNFVGQK-----------KQLEAEISNTRNELTNLD-----------------------------------KSKLELNNSISILTAEKRRLELNCTVS----------RSEIIQLQTQID----ELQQE-------KQEIENSLTLLGR   220
Pleurocapsales_cyanobacterium_LEGE_06147 -----SQK-----------QQLETEYNNWQAQVREVF-----------------------------------QQQEQTNQTLSSLETQKEAIETSIATR----------QTELEQIRDRLM----QAQQQ-------QEELDTYLARLES   196
Anabaenopsis_elenkinii_CCIBt3563         IGIFASQK-----------KQLETDTINLTAQIESLE-----------------------------------QNKIELHHTCSQMTTEKRRLELNCNSF----------RAEIMQLQNQIS----QLQEE-------KKELENNVTLLDR   202
                                         .......160.......170.......180.......190.......200.......210.......220.......230.......240.......250.......260.......270.......280.......290.......300
```

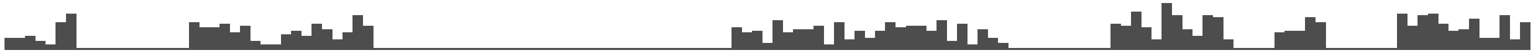

SepT MULTIPLE SEQUENCE ALIGNMENT


```
                                          : ::.     ::           .       :                       .                                                                                       .  . .
Nostoc_sp._PCC_7120_=_FACHB-418          IKPQLEEKLYELRINIQDLELEKSKQSQI---LLDTS--------------NEQESLA-----------------------------------------ANIDYLQIQKTER-----------HNELQQIQNEIFLLQQERDILQNQVWE   283
Nostoc_punctiforme_PCC_73102             LKPQLEEKLYELRIAIQELEVETTQKNQL---LVATK--------------TERENIQ-----------------------------------------AILHSSQTKLVEH-----------KSELQQLQGQISLLQEERDSLQNQVWE   283
Nostoc_azollae_0708                      LKPQLDEKLYELRIEIHELEIKVSNQNNL---LTHTI--------------TARENLE-----------------------------------------NILTDLYTKKQAQ-----------QSEISQLHNQISLLQDERHLLQNQVWE   301
Fischerella_thermalis                    LKPQIEEKLYEMRLQVQELEAANQQQNQL---LLDKK--------------LEAENIE-----------------------------------------INLNSLKAEVAEQ-----------QNQIQQLQSQISLLQDERDHLQSQVWE   289
Tolypothrix_sp._PCC_7601                 LKPQIEEKLYELRIELQSLEVEFNQKNQL---LLNTS--------------NNPENVE-----------------------------------------SNLNDVQTKIGEH-----------QVELQKLQAQIFLLQEERDLLQSQVWE   283
Spirulina_subsalsa_PCC_9445              QKYKLSQELQTLKDELKDLQNFQEKLSQS---LAELELQREYNDEQIFYKEARREGLELEIENLEKILLEKQELWQNLEPKMLKWERLQAEGVKLEENQSSLTAELHQLAEDKHRLSLELNQLRQNQSTLTVELNQLAQEKQSLSAELNQ   356
Mastigocladopsis_repens_PCC_10914        LKPQLEEKLYELRVQIQDLEVQGTQQKQL---IVEKK--------------AEKELIE-----------------------------------------ANLNSLQTQLAEQ-----------RTHLQQLQEQVTLLQEERDQLQSQVWE   289
Rivularia_sp._PCC_7116                   LKPQLEERLYELRVQVEELETEQKKQQEL---FSSRN--------------KKQEQLK-----------------------------------------ADIKLLKNQKAEE-----------KKQLEELQGQTSLLQEERDVLQNQVLE   294
Chlorogloeopsis_fritschii_PCC_9212       LKPQLEEKLYQMRVKIHELETEEKRLSQI---LIDGK--------------NETENVE-----------------------------------------SNLNSLRTQVAEH-----------ETQLHHLQEQVVLLQDERDNLQSQVWE   283
Chlorogloeopsis_fritschii_PCC_6912       LKPQLEEKLYQMRVKIHELETEEKRLSQI---LIDGK--------------NETENVE-----------------------------------------SNLNSLRTQVAEH-----------ETQLHHLQEQVVLLQDERDNLQSQVWE   283
Microcoleus_sp._PCC_7113                 QKRNLEGEAYDLQTQIQVLQQRQEQLNQV---LAPLQ--------------EQQQQVEV----------------------------------SLMAGKAKLDQLLSQISER-----------RKQQKKLNQDLANLESRKQQLEAEFQH   297
Gloeocapsa_sp._PCC_7428                  QKRQIELKTYILQNQIEKLEQYKNNIQNK---LTSLK--------------GEKEEVR-----------------------------------------ASLETLQLKKEQQ------------------QADLLALQEERNQLQSQILE   335
Nodularia_spumigena_CCY9414              LKPQLESKMYELRIETQELEAETNQQKEF---LVSTK--------------TERENLA-----------------------------------------ENLNYLENKITEK-----------HSELQQIEAQVSLLQSERDLLQSQVWE   283
Scytonema_hofmannii_PCC_7110             LKPQLEEKLYELRSEIHEMEVQEQQQSQV---LTAKK--------------AERESIE-----------------------------------------ASLNSLHAELMEQ-----------RKELEQLQGQVSLLLEERDQLQNQVWE   289
Richelia_intracellularis_HH01            LRPQLEEKLYEMRLQLQELELQESKKNEI---LKVND--------------EAKNILE-----------------------------------------MSSTSLDDTIAQQ-----------KAEFRQLQEQITILQTERDQLQKEIWE   276
Aphanizomenon_flos-aquae_NIES-81         LKPQLEEKLYELRIEIQNQELEITKQNNL---LVETG--------------ITKNNLE-----------------------------------------NTLKSLQNKTLEQ-----------KTEVNQLQNQISVLQDERDLLQNQVWE   301
Aphanizomenon_flos-aquae_2012/KM1/D3     LKPQLEEKLYELRIEIHNQELEITKQNNL---LVETG--------------ITRNNLE-----------------------------------------NTLKSLQNKTLEQ-----------KMEVNQLQNQISVLQDERDLLQNQVWE   301
Spirulina_major_PCC_6313                 RRDELSDTRQHLQTQQAELLTLHQEQETLQARLRTLQ--------------AEHDTLI-----------------------------------------PQLTTLQHQHQDL-----------SGQVQQQQAQLAIAQQDYTTAQNQLQD   278
Nodularia_sp._NIES-3585                  LKPQLEEKMYELRIEVQELEAETNQQKQL---LVSTK--------------TERENLA-----------------------------------------ENINYLQTKITEK-----------QSELHQIESQISLLQEERDLFQNQIWE   283
Anabaenopsis_circularis_NIES-21          LKPQLEEKMYELRIQIQELEIVVNQQNKL---LADTI--------------TERNDII-----------------------------------------SRLNNLTQQTSDK-----------ESELQQLNSQVSILQEERDLLQNQVWE   283
Calothrix_sp._NIES-2098                  LKPQVEEKLYELRIQLQDLELEVNQKNQL---LSNTT--------------NNRENIE-----------------------------------------SRLNDLQTKTIEK-----------QAELHQLEEQVSLLQSERDLLQSQVWD   283
Chroococcidiopsis_sp._TS-821             QKRQIELKTYIIQNQVEKLEHHKKELQEK---LNSLK--------------TEKEEVR-----------------------------------------AILEALQLQKEQQ------------------QTELLALQEERNQLQSQILE   290
Calothrix_desertica_PCC_7102             LKPQLEEKLHELRVQIQELEVKENHKNSI---ISEKS--------------NERKTLD-----------------------------------------SKIKSLQTKLTTK-----------QEELKTLQDQVSLLQDERNLLQTQVWE   280
Cylindrospermum_sp._NIES-4074            LKPQLEEKLYELRIEIQELEIYTAKQNQL---LATTK--------------NERADIE-----------------------------------------SSLNSLQTQTTTQ-----------QAELNQLQEQVKLLQDERDLLQSQVWE   301
Pleurocapsales_cyanobacterium_LEGE_06147 RKQELETENQNLQAQIQELEQQHAQLAQV---IAPLA--EEP---------AESETFE-------------------------------------DRSNEELEELIGLEELD--------N--FSKEELVSVAVKLTDEESISVENPMDE   285
Anabaenopsis_elenkinii_CCIBt3563         LKPQLEEKMYELRTEIQALETQINEQKQL---LLSTK--------------TETENLA-----------------------------------------ENLNYLESQINQK-----------QSELQQITSELSLLQAERDSLQSQVWE   283
                                         .......310.......320.......330.......340.......350.......360.......370.......380.......390.......400.......410.......420.......430.......440.......450
```

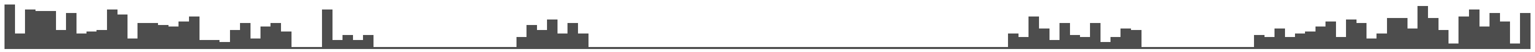

```
                                                                                                          :          .   :   :   :      ::         :              : ::  *   : .             .:
Nostoc_sp._PCC_7120_=_FACHB-418          LLQ---QIETV-NQE--PISINTS-------EQEGTFFSFSD-ILENLDKI-DAKNDTSQ----ALPE--------EWHTLLKQLPSHEIQVLKIIIGQDNPQASIKKIAEANITMPNLLIDSINERANDTIGEL------------IIE   394
Nostoc_punctiforme_PCC_73102             LLQ---QLETF-NQE--PLADSFQ-------EDDTELFPFSE-IMETTAVIGNSEIDTSE----NIPE--------EWTNFLENLPGYELQVLKAIVEQDNPKAAIKKIAEANITMPNLLIDSINERANDTIGEL------------IIN   395
Nostoc_azollae_0708                      LLQ---NMETL-DQD--ALTENTQ--------EDLELFPFDE-ILEPV----DPSSPTSD----NLPP--------EWSNFLEKLPTNQIHVLKAIVAQDNPKAIIKQIAEEHMTMPNLLIDAINEVANLTLGEL------------VIK   408
Fischerella_thermalis                    FLQ---QIENL-DSN--NVLEKNQ-------EEDVDVFPFAD-LLDTVNDI-DG-SKILE----GLSS--------EWTNFLAQMSDSEIQALKAMIEDENPHSVIKKIAEANITMPNLLIDSINEKANNTIGEL------------IID   399
Tolypothrix_sp._PCC_7601                 LLQ---QLETL-NPE--PLADRRH-------EENVELFPFAE-LIEPLETI-DIKPDTSE----ALSE--------EWYHFFEHLQNHEIDVIKALLSAENPQTTIKQIAEVNITMPNLLIDAINGKASDNLGEL------------IIN   394
Spirulina_subsalsa_PCC_9445              LRQ---NQSTL-TTELNQLAQEKQ-------NLSAELATLEDQLTTPIRTP--STPSKPA----PFPE--------EWLDFLHNLCGSEKQTLKAILDQD--EERLKQLADEVTTMPEVLIDALNEKALNTIGDT------------FLI   467
Mastigocladopsis_repens_PCC_10914        LLY---QMETLTTQE--QLNENRL-------EDSEEFFPFSE-LVEFLEPI-ET-ADQLE----NFSE--------EWTEFVEQLHGYEIQVLKAILEQNNPNAAIKKIAEENITMPNLLIDAINERANDTIGEL------------IID   400
Rivularia_sp._PCC_7116                   LLQ---QLEGL-NPE--LTISQAE------AEEETEEFPFAD-LIESISIE-----PASD----ELPQ--------EWKDLQQSLLEHEIKVLKAILEQVKVNSKIKKIAEQNISMPNLLIDSINERAQDTIGEL------------IIA   402
Chlorogloeopsis_fritschii_PCC_9212       LLQ---QTEVL-HHD--NLLEQGQ-------EEEVDLFPFAE-IIEPVENN-NSSSEIVE----IIPE--------EWVKFIEKLPAHEIQVLKAIIEQENPYTAIKKIAEDNITMPNLLIDSINERANDTLGEL------------IID   394
Chlorogloeopsis_fritschii_PCC_6912       LLQ---QTEVL-HHD--NLLEQGQ-------EEEVDLFPFAE-IIEPVENN-NSSSEIVE----IIPE--------EWVKFIEKLPAHEIQVLKAIIEQENPYTAIKKIAEDNITMPNLLIDSINERANDTLGEL------------IID   394
Microcoleus_sp._PCC_7113                 WQT---QIQVL-EPQ--RTA------------------------RSPIYTL-DSDSVISQ----ILPEEW-----HEWLEFIQQLSNQEQRALKAILEQD--RATLKRIADENSTMPEVLIDSINENALNIFGDT------------LFV   393
Gloeocapsa_sp._PCC_7428                  FHQ---QIEAL-IAE--PISDDDK-------QEDTELFPFSE-LIDSLEIP-EVEVTISH----ELPE--------EWMEFMEQLPGYQVQILKAILEQNHPSKIIKEIAESKVTMPTILIDNINECAIDTIGDR------------IID   446
Nodularia_spumigena_CCY9414              LLQ---QTETL-NPE--ASPVNLN-------QDANDVFPFAE-LIDSLDSI-----DKYE----ILPE--------EWNNFLGVLPNAEIHVLKAIVEQDNPQVIIKQIAEANITMPNLLIDSINEHANDTIGEL------------IIE   390
Scytonema_hofmannii_PCC_7110             LLQ---QMETL-TSD--PLFDNNE------QDREAEFFPFSE-LMDSLEPV-NILADNSE----DLSE--------EWAKFVEQLPNYEIQVLKAILEQENPNPTIKKIAEDNITMPNLLIDSINELARDTIGEL------------LID   401
Richelia_intracellularis_HH01            LLQ---QFDNV-AQT--PLDEQ--------VDSEMELFPFAE-LIQEVNDM-----EIVD----ELPP--------EWNLLLQNLPPNEIEVLKIILQEDSNNEIDEKSPEQELNMPSLLINSINKRANNIFGKL------------IIE   382
Aphanizomenon_flos-aquae_NIES-81         LLQ---QVETI-NPS--TLPDN-E-------ENNLDLFPFDE-LLEPLDPI-NGKPDHTK----NLPA--------KWNNFFNHLPGYEIQVLKAILEQDNPKATIKEIAEANITMPNLLIDAINEIASNTIGEL------------IIE   411
Aphanizomenon_flos-aquae_2012/KM1/D3     LLQ---QVETI-NPS--TLPDN-E-------ENNLDLFPFDE-LLEPLDPI-NGKHDHTE----NLPA--------KWNNFFNHLPGYEIQVLKAILEQDNPKATIKEIAEANITMPNLLIDAINEIASNTIGEL------------IIE   411
Spirulina_major_PCC_6313                 TLD---TLTTT-QNQ--LTALETQ---------------LAE-LQQPEL------PGRVY----ALAS--------EWYEFVHALTSLEKRALVAILDQD--EAQIKTLCRAADLDVPALVESLNETALMALGDL------------LLD   374
Nodularia_sp._NIES-3585                  LLQ---QTETL-NQQ--VAPKNLN-------EDANNLFSFPE-LIDSLDSI-----ETSD----ILPK--------EWNNLLKILPIHEIQVLKAIVEQDKPQVIIKKIAEANITMPNLLIDSINEHANDTIGEL------------IIE   390
Anabaenopsis_circularis_NIES-21          LLQ---QTETL-NLE--HIPINLP------IEDEPELFPFAD-LIATPE-------DISE----DLPE--------EWTNLLEKLPGHEIEVLKAILQADNPKATIKQIAEANITMPNLLIDSINDRANDTIGEL------------IIE   389
Calothrix_sp._NIES-2098                  LLQ---QIETL-NPE--IFPEKVS-------EDIQEIFPFEE-LIQPIEIF-NIETDVSE----NLPE--------EWNNFLHQLPDYEIQVLKAILEQENSRDVIKQIAEANITMPNLLIDSINDKADATLGEL------------IID   394
Chroococcidiopsis_sp._TS-821             FHQ---QIEAL-IAE--PVS-DNH-------QEDTE-FPFLE-LIDSLETP-DIEVDLSP----ELAP--------EWNEFMEQLPGYQIQILKVILEQNNPNQFIKEVAESKLTMPALLIDNINERAINTIGDR------------IIN   399
Calothrix_desertica_PCC_7102             LLQ---SAEKL-NPE--YSIEENHNIYDNGVEAEAE-FPFSD-LLGIIEPV-STQNIQSQNQIVELSG--------EWDNFRRQLNQSEFLVLKAILEQENPNASIKKIAEENITMPSLLIDAVNELASNTIGEL------------IIE   401
Cylindrospermum_sp._NIES-4074            LLQ---QAETF-NQE--SLPETAE-------EDDIDLFPFSE-LMDSLEPR-NSHLDTAQ----NLPE--------EWANFLENLPAYELQVLKAIVEEDNPKPAIKQIAEEHITMPNLLIDSINELANDTIGEL------------IIE   412
Pleurocapsales_cyanobacterium_LEGE_06147 MEEIAFQEEPIFLEDLDSIDEQES------VNEESE--PFLS-LIEL-----ESTPENSK----ELDNLFDGNPIADDDSFREELAQPESTTEDFVLLDESIEPSEEIACEEPISLEEL--DSIDEEAIASMTEFESAAENSEGLDNLLD   415
Anabaenopsis_elenkinii_CCIBt3563         LLQ---QPETL-NPE--LSPENCH-------EDGNDVFPFTE-LIDSVKTI-----NTSE----ILPE--------QWQKLLEILPNHEIQALKAIVEQEHPLVTIEEIAQANSTTPSLLIDSLNEHAQDAIGEL------------IIT   390
                                         .......460.......470.......480.......490.......500.......510.......520.......530.......540.......550.......560.......570.......580.......590.......600
```

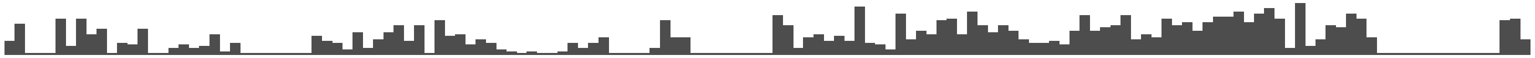

SepT MULTIPLE SEQUENCE ALIGNMENT


```
                                                                                  :        :
Nostoc_sp._PCC_7120_=_FACHB-418          TDL-------------------------------DVPKIYAEHITNVRNIVA--------------------------------------------------------------------------------------------------    415
Nostoc_punctiforme_PCC_73102             SDS-------------------------------ESPEVYHEHITYVKKMIV--------------------------------------------------------------------------------------------------    416
Nostoc_azollae_0708                      TDA-------------------------------EIPEIYQDHILNLRKMLT--------------------------------------------------------------------------------------------------    429
Fischerella_thermalis                    PNA-------------------------------TIPEIYPEYQSDVKRAIA--------------------------------------------------------------------------------------------------    420
Tolypothrix_sp._PCC_7601                 TNV-------------------------------EPPEIYPEYISNMKKMLA--------------------------------------------------------------------------------------------------    415
Spirulina_subsalsa_PCC_9445              RQNL-----------------------------AILPTIDEEYHSLFSQPLSL-------------------------------------------------------------------------------------------------    491
Mastigocladopsis_repens_PCC_10914        PSS-------------------------------ENPEIHQEYMTNVKKVIA--------------------------------------------------------------------------------------------------    421
Rivularia_sp._PCC_7116                   TSN-------------------------------DIPELYDEYKENIQKIIA--------------------------------------------------------------------------------------------------    423
Chlorogloeopsis_fritschii_PCC_9212       PSL-------------------------------DVPQIYAEHQSNVKRAIA--------------------------------------------------------------------------------------------------    415
Chlorogloeopsis_fritschii_PCC_6912       PSL-------------------------------DVPQIYAEHQSNVKRAIA--------------------------------------------------------------------------------------------------    415
Microcoleus_sp._PCC_7113                 SGSI-----------------------------SLIPEIHEDYSLILKEPIIL-------------------------------------------------------------------------------------------------    417
Gloeocapsa_sp._PCC_7428                  FDS-------------------------------EQAEIVEDYRENVKQVIQ--------------------------------------------------------------------------------------------------    467
Nodularia_spumigena_CCY9414              TNL-------------------------------ENPQVYPEHIVNVRKMLAC--------------------------------------------------------------------------------------------------    412
Scytonema_hofmannii_PCC_7110             TAS-------------------------------DNPAIYQEYIRDVRKAIA--------------------------------------------------------------------------------------------------    422
Richelia_intracellularis_HH01            LEH-------------------------------ESPIISPEHRNNVSKLVH--------------------------------------------------------------------------------------------------    403
Aphanizomenon_flos-aquae_NIES-81         TNT-------------------------------EVPEIVEEHLLNVKTMIT--------------------------------------------------------------------------------------------------    432
Aphanizomenon_flos-aquae_2012/KM1/D3     TNT-------------------------------EVPEIVEEHLLNVKTMIT--------------------------------------------------------------------------------------------------    432
Spirulina_major_PCC_6313                 FDTT-----------------------------TDLLTMNEDYAMIMAGSVAV-------------------------------------------------------------------------------------------------    398
Nodularia_sp._NIES-3585                  PSL-------------------------------EIPEVYEEHIVNARKMLA--------------------------------------------------------------------------------------------------    411
Anabaenopsis_circularis_NIES-21          PGE-------------------------------DIPEIYQEHLNNVRKMIA--------------------------------------------------------------------------------------------------    410
Calothrix_sp._NIES-2098                  PRV-------------------------------ENPEIYHEHTNNVKKMLA--------------------------------------------------------------------------------------------------    415
Chroococcidiopsis_sp._TS-821             FDS-------------------------------KQVEIIEDYRENVKQVIQ--------------------------------------------------------------------------------------------------    420
Calothrix_desertica_PCC_7102             TTS-------------------------------EYPKIYTEHIENVCIIIK--------------------------------------------------------------------------------------------------    422
Cylindrospermum_sp._NIES-4074            TSA-------------------------------EIPAVYQEHISNVKKMIS--------------------------------------------------------------------------------------------------    433
Pleurocapsales_cyanobacterium_LEGE_06147 STNTFDSQFLEQLMDETAELSILDLDLVEELAQPKSPESTTEESVTLENLMSEAEIASPVKSRAAQTELTNELDDHFFSFINEATEEETTNTGQESEQLDFLISSSYTSESRELEITSTESGFLENSIDRAEENFLENFESLEKLPETEI    565
Anabaenopsis_elenkinii_CCIBt3563         TNG-------------------------------EHPQVSEEHIVNARKILA--------------------------------------------------------------------------------------------------    411
                                         .......610.......620.......630.......640.......650.......660.......670.......680.......690.......700.......710.......720.......730.......740.......750
```

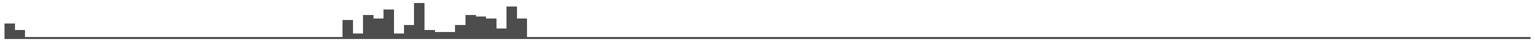

```
Nostoc_sp._PCC_7120_=_FACHB-418          --------------------------------------------------------------------LYEHLIARYAS----------------------------------    426
Nostoc_punctiforme_PCC_73102             --------------------------------------------------------------------MHESLMARHTSPN--------------------------------    429
Nostoc_azollae_0708                      --------------------------------------------------------------------KHEELITQS------------------------------------    438
Fischerella_thermalis                    --------------------------------------------------------------------TYKTIN---------------------------------------    426
Tolypothrix_sp._PCC_7601                 --------------------------------------------------------------------NYQEDI---------------------------------------    421
Spirulina_subsalsa_PCC_9445              --------------------------------------------------------------------QFQDLLTLIHSTHA-----------ENT-----------------    508
Mastigocladopsis_repens_PCC_10914        --------------------------------------------------------------------MYEDKMAGQISSK--------------------------------    434
Rivularia_sp._PCC_7116                   --------------------------------------------------------------------MSEGVESK-------------------------------------    431
Chlorogloeopsis_fritschii_PCC_9212       --------------------------------------------------------------------VHQDMMARLTSSN--------------------------------    428
Chlorogloeopsis_fritschii_PCC_6912       --------------------------------------------------------------------VHQDMMARLTSSN--------------------------------    428
Microcoleus_sp._PCC_7113                 --------------------------------------------------------------------YFKDLLALQSAPFKI---SDAI--TENSDTSSSSREIIVPPQSKT    457
Gloeocapsa_sp._PCC_7428                  --------------------------------------------------------------------MYEGIMSS-------------------------------------    475
Nodularia_spumigena_CCY9414              -------------------------------------------------------------------DIY-------------------------------------------    415
Scytonema_hofmannii_PCC_7110             --------------------------------------------------------------------IHQDIMARQTSRN--------------------------------    435
Richelia_intracellularis_HH01            --------------------------------------------------------------------TYDILSSKQSSSN--------------------------------    416
Aphanizomenon_flos-aquae_NIES-81         --------------------------------------------------------------------KYSEIS---------------------------------------    438
Aphanizomenon_flos-aquae_2012/KM1/D3     --------------------------------------------------------------------KYSEIS---------------------------------------    438
Spirulina_major_PCC_6313                 --------------------------------------------------------------------LFRELLTIGVEG---------------------------------    410
Nodularia_sp._NIES-3585                  --------------------------------------------------------------------IYENLINRQASSN--------------------------------    424
Anabaenopsis_circularis_NIES-21          --------------------------------------------------------------------MYEDLMSRQASSN--------------------------------    423
Calothrix_sp._NIES-2098                  --------------------------------------------------------------------IYQHSS---------------------------------------    421
Chroococcidiopsis_sp._TS-821             --------------------------------------------------------------------VYEGIMGS-------------------------------------    428
Calothrix_desertica_PCC_7102             --------------------------------------------------------------------AYETAATETHFA---------------------------------    434
Cylindrospermum_sp._NIES-4074            --------------------------------------------------------------------MYG------------------------------------------    436
Pleurocapsales_cyanobacterium_LEGE_06147 DESSTIDHFLTENLDLENSFDRWEETSEELGQLDDLSTSMSEYSMQGKLEEVEEKLCELNSISEASLEEFEDLLESMEEQNSSISFSDDVDDIETTKTQNLWD----------    668
Anabaenopsis_elenkinii_CCIBt3563         --------------------------------------------------------------------MAVDVFSSL------------------------------------    420
                                         .......760.......770.......780.......790.......800.......810.......820.......830.......840.......850.......860...
```

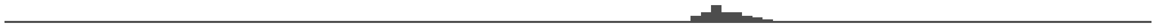

Fig. S1. Amino acid sequence alignment used as input for the SepT phylogenetic tree reconstruction (see Materials and methods for details). The alignment was depicted using ClustalX.
