# Supplementary material for: SepT, a novel protein specific to multicellular cyanobacteria, influences peptidoglycan growth and septal nanopore formation in Anabaena sp. PCC 7120
Source: mBio. 2023 Aug 31;14(5):e00983-23. doi: 10.1128/mbio.00983-23 (PMC10653889; doi:10.1128/mbio.00983-23)
Supplement: Fig. S2 — Localization of truncated SepT-GFP in Anabaena. [file mbio.00983-23-s0005.pdf]

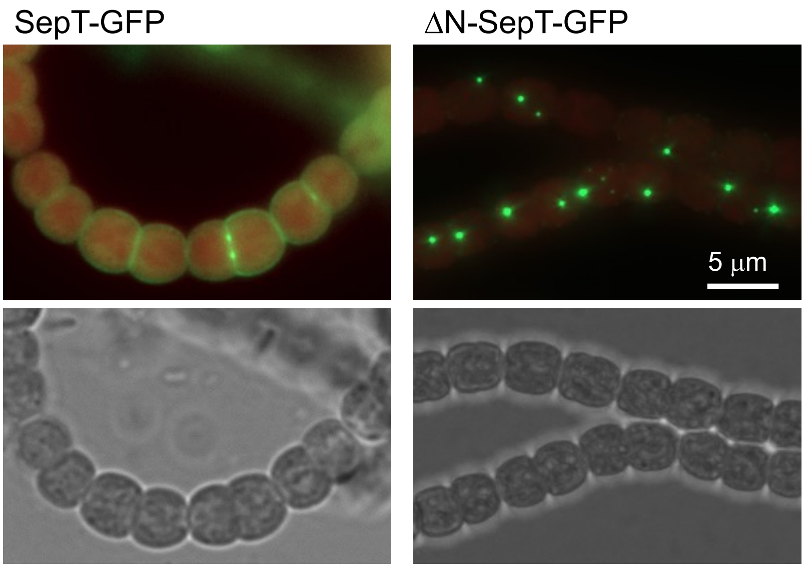

Fig S2. Localization of truncated SepT-GFP in *Anabaena*. Filaments of *Anabaena* derivatives expressing SepT-GFP or a SepT-GFP version without the N-terminal transmembrane domain of SepT (first 52 aa removed; ΔN-SepT) from P_{petE}, were grown in solid medium with nitrate. The strain expressing ΔN-SepT was further incubated for 24 h in liquid medium supplemented with 0.2 μM $CuSO_4$, as no fluorescence signal could be detected otherwise. Filaments were observed under a fluorescence microscope and photographed. Merged GFP-fluorescence (green) and chlorophyll autofluorescence (red) and bright field micrographs are shown. Magnification is the same for all micrographs.
