# Supplementary material for: SepT, a novel protein specific to multicellular cyanobacteria, influences peptidoglycan growth and septal nanopore formation in Anabaena sp. PCC 7120
Source: mBio. 2023 Aug 31;14(5):e00983-23. doi: 10.1128/mbio.00983-23 (PMC10653889; doi:10.1128/mbio.00983-23)
Supplement: Fig. S3 — Septal nanopore array in strain BS1 grown on solid medium. [file mbio.00983-23-s0006.pdf]

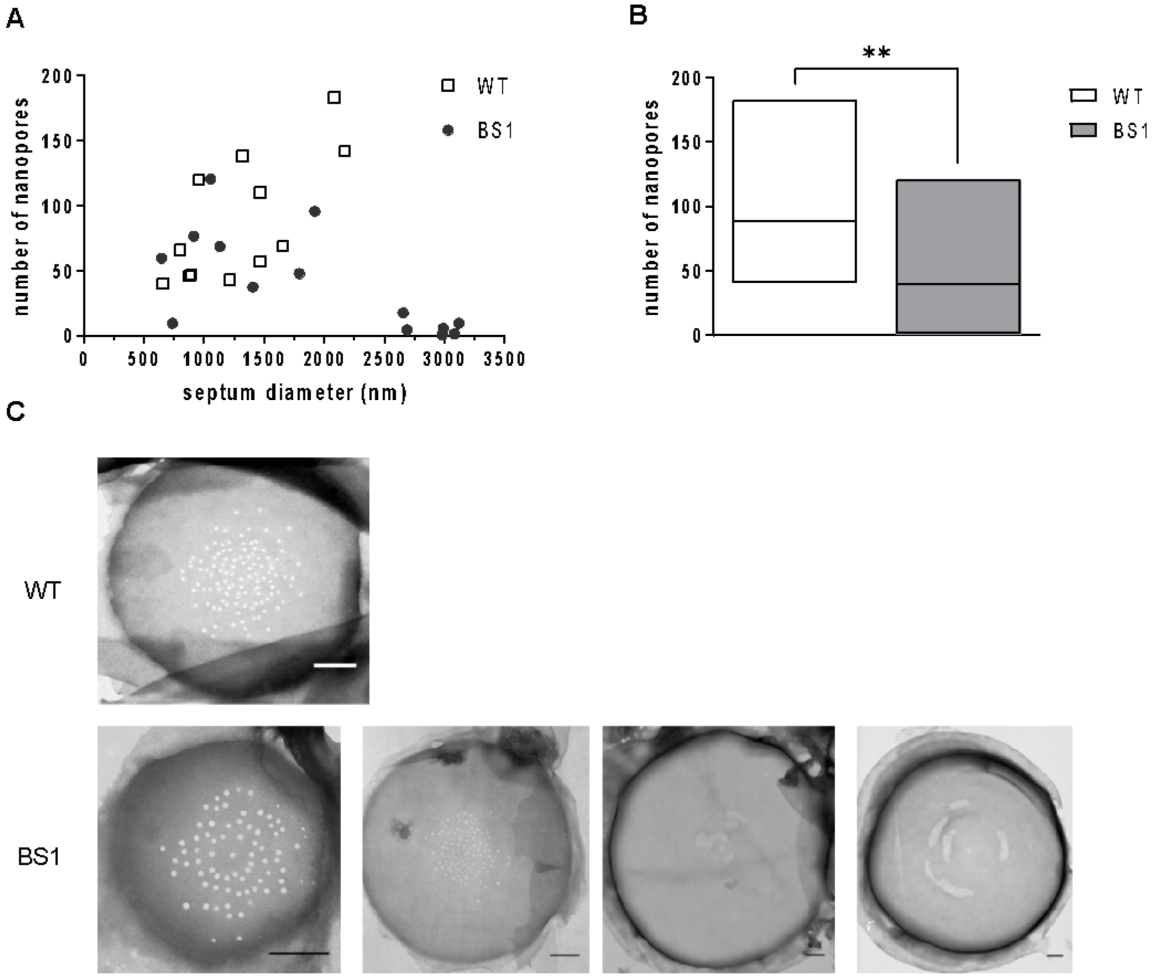

Figure S3. Septal nanopore array in strain BS1 grown on solid medium. Peptidoglycan was purified from filaments of the wild type (WT) and mutant BS1 that were grown on agar plates, and the sacculi investigated by TEM. A, The number of nanopores is shown correlated to the septum size. In contrast to the WT, the mutant shows a subset of large septa with very few nanopores. B, The number of pores per septum was analyzed. Floating bars show the mean value. Unpaired Student‘s *t*-test was performed (mutant vs. WT). *p*-values are indicated (**p<0.001). C, Representative TEM images of indicated strains are shown. Bars: 250 nm.

Methods: Transmission electron microscopy and sacculi preparation. Peptidoglycan sacculi were isolated from filaments grown on BG11 agar plates, by the method of Kühner *et al.* (2014) with the following modifications: cells were sonicated (Branson Sonifier 250; duty cycle 50%, output control 1, 2 min) prior to boiling in 1 mL 0.1 M Tris/HCl pH 6.8 with 3% SDS. After the sonifier waterbath, the sample was incubated with 600 µg α-chymotrypsin at 37° C overnight in 50 mM Na_3PO_4 buffer pH 6.8. After inactivation of the enzyme, the sample was sonified again and loaded on a formvar/carbon film coated copper grid (Science Services GmbH München) and stained with 1 % (w/v) uranyl acetate as described previously (Lehner *et al.*, 2011). Images were taken with a Philips Tecnai10 electron microscope at 80 kV.

References:

Kühner D, Stahl M, Demircioglu DD, Bertsche U. 2014. From cells to muropeptide structures in 24 h: Peptidoglycan mapping by UPLC-MS. Protocol Exchange.

Lehner J, Zhang Y, Berendt S, Rasse TM, Forchhammer K, Maldener I. 2011. The morphogene AmiC2 is pivotal for multicellular development in the cyanobacterium *Nostoc punctiforme*. Mol Microbiol 79:1655-1669.
