# Supplementary material for: SepT, a novel protein specific to multicellular cyanobacteria, influences peptidoglycan growth and septal nanopore formation in Anabaena sp. PCC 7120
Source: mBio. 2023 Aug 31;14(5):e00983-23. doi: 10.1128/mbio.00983-23 (PMC10653889; doi:10.1128/mbio.00983-23)
Supplement: Table S1 — Cyanobacterial strains and plasmids used in this work. [file mbio.00983-23-s0007.pdf]

Table S1. Cyanobacterial strains and plasmids used in this work.

**Strain**	**Genotype**	**Resistance**	**Source**
Anabaena sp. PCC 7120	WT		Pasteur Culture Collection
BS1	*sepT*::C.S3	Sm, Sp	This study
CSCV6	*thrS2*::P_{mreB}-*sfgfp-mreB*	Sm, Sp	Velázquez-Suárez *et al.*, 2022a
CSCV7	*thrS2*::P_{mreB}-*sfgfp-mreC*	Sm, Sp	Velázquez-Suárez *et al.*, 2022a
CSCV8	*thrS2*::P_{mreB}-*sfgfp-mreD*	Sm, Sp	Velázquez-Suárez *et al.*, 2022a
CSCV9	*sepT*::C.K1	Nm	This study
CSCV11	*thrS2*::P_{mreB}-*sfgfp-mreB*, *sepT*(CSCV9)	Nm, Sm, Sp	This study
CSCV12	*thrS2*::P_{mreB}-*sfgfp-mreC*, *sepT*(CSCV9)	Nm, Sm, Sp	This study
CSCV13	*thrS2*::P_{mreB}-*sfgfp-mreD*, *sepT*(CSCV9)	Nm, Sm, Sp	This study
CSCV25	P_{sepT}-*sepT-gfpmut3.1*	Nm	This study
CSCV26	P_{sepT}-*sepT-gfpmut3.1*, *sepT*(BS1)	Nm, Sm, Sp	This study

**Plasmid**	**Description**	**Resistance marker**	**Source**
pCSCV36	pRL277 carrying *sepT*::C.K1	Km, Nm	This study
pTHS109	pRL278 carrying *sepT*::C.S3	Sm, Sp	This study
pTHS240	pRL25C carrying P_{sepT}-*sepT-gfpmut3.1*	Km, Nm	This study
pTHS143	pRL25C carrying P_{petE}-*sepT-gfpmut3.1*	Km, Nm	This study
