# Supplementary material for: SepT, a novel protein specific to multicellular cyanobacteria, influences peptidoglycan growth and septal nanopore formation in Anabaena sp. PCC 7120
Source: mBio. 2023 Aug 31;14(5):e00983-23. doi: 10.1128/mbio.00983-23 (PMC10653889; doi:10.1128/mbio.00983-23)
Supplement: Table S2 — Oligodeoxynucleotide primers used in this work. [file mbio.00983-23-s0008.pdf]

Table S2. Oligodeoxynucleotide primers used in this work.

Name	Sequence (5'-3')
842KO_2A	ATTCGATATCTAGATCTCGAGATGGATAATCCAGCAATGTCGGC
842KO_2B	AAGGTGCTGTGCACGGATCATTGCTGATTTTTAGCGTAGTTAAGCTT T
842KO_4A	CAAGGTAGTCGGCAAATAAAATTTAATATCCCTAGCTCATCGTAAA ATTTTTATAAAAATATG
842KO_4B	ATGCAAGCTTTCGCGAGCTCTTTAAAACTAGAACTATGAACTAGCT CGCTAAAC
all2459-1	AATGCTGCAGCACCTTCGGCAATATCATC
all2460-1	GAATGGATCCACGGAGTGAACTTAAAAG
all2460-2	AATTGGATCCGCTAACATCACTATGCCG
all2461-1	CTTTCTGCAGCGCTTTGGCTTAACCCTC
alr0653-1	CAACTCCTGCAGGGTTATTAAAACGATCGCTTC
alr0653-2	GATGACGAATTCTAGTGGTTGGTGGTCAGT
alr0653-3	CAAAACTGCAGCTTATTAAAACGATCGCTTC
alr0653-4	GAAAGTGAATTCGTAAAATAATATTTCTGTCGTTGTC
alr5045-7	TTTAAGCTGCAGGGCTTTACTGCAACCATC
alr5045-8	TATTTAGAATTCATATTATCTGCTTTTTGC
alr5045-9	CTTTAACTGCAGTGGCTTTACTGCAACCAT
CS.3_Fwd	GATCCGTGCACAGCACCTTG
CS.3_Rev	TTATTTGCCGACTACCTTGGTGATCT
GFP_25C_R	AGGCCCTTTCGTCTTCAAGTTATTTGTATAGTTCATCCATGCCATGT GT
GFP_842_A	AGCTAGATACGCATCCGCTAGTGCATCTGCTAGTGCTAGTG
MB_25A	TGCCTGCAGGTCGACTCTAATGCAACAAGTCATAGTAAGTAATCGA T
MB_25B	TCGGTACCCGGGGATCCTCGGATGCGTATCTAGCTATTAGATGTTC
MB_26A	AGGGTCGACTCTAGAGGATATGCAACAAGTCATAGTAAGTAATCG AT
MB_26B	CTTACTTAGGTACCCGGGGGGATGCGTATCTAGCTATTAGATGTTC
MB_27A	TGCCTGCAGGTCGACTCTAATGCAACAAGTCATAGTAAGTAATCGA T
MB_27B	TCGGTACCCGGGGATCCTCGGATGCGTATCTAGCTATTAGATGTTC
MB_28A	TCTAGAGGATCCCCGGGTAATGCAACAAGTCATAGTAAGTAATCGA T
MB_28B	TCGATGAATTCGAGCTCGGGGATGCGTATCTAGCTATTAGATGTTC
Nos842_2A	TACAGGTTAGGAGAACGCCATGCAACAAGTCATAGTAAGTAATCG ATT
Nos842_2B	CACTAGCAGATGCACTAGCGGATGCGTATCTAGCTATTAGATGTTC
p842_25C_lo ng_A	TTTTGGTCATGAGATTATCAAAAAGTCTCTCTATCCCCAAGTACAAT TTCTCC
petE_903_Fw d	GAGATTATCAAAAAGGATCCCAGTACTCAGAATTTTTTGCTGAGGT ACT
pRL25C_F	CTTGAAGACGAAAGGGCCTCT
pRL25c_NEB _Fwd	GCTAGTGCATCTGCTAGTGCTAGTG
pRL25c_NEB _Rev	GGCGTTCTCCTAACCTGTAGTTTTATTTTTCT
pRL25C_R	CTTTTTGATAATCTCATGACCAAAATCCCTTAAC
pRL25c-903_V_F	TGGATGAACTATACAAATAAAGAATTCTTGAAGACGAAAGGGCC
pRL25c-	GCAAAAAATTCTGAGTACTGGGATCCTTTTTGATAATCTCATGACC

903_V_R	AAAATCC
pRL271_Fwd	GAGCTCGCGAAAGCTTGCATG
pRL271_Rev	CTCGAGATCTAGATATCGAATTTCTGCCAT
